# Validation of neuromuscular blocking agent use in acute respiratory distress syndrome: a meta-analysis of randomized trials

**DOI:** 10.1186/s13054-020-2765-2

**Published:** 2020-02-17

**Authors:** Wei Chang, Qin Sun, Fei Peng, Jianfeng Xie, Haibo Qiu, Yi Yang

**Affiliations:** Department of Critical Care Medicine, Zhongda Hospital, School of Medicine, Southeast University, Nanjing, 210009 China

**Keywords:** Acute respiratory distress syndrome, Neuromuscular blocking agent, ARDS, NMBA, Mortality

## Abstract

**Background:**

We aimed to synthesize up-to-date trials to validate the effects of neuromuscular blocking agent (NMBA) use in patients with moderate-to-severe acute respiratory distress syndrome (ARDS).

**Methods:**

Several databases including PubMed, EMBASE, Web of Science, and Cochrane Central Register were searched up to November 14, 2019. All randomized trials investigating the use of NMBAs in patients with moderate-to-severe ARDS and reporting mortality data were included in the meta-analysis. The primary outcome was mortality, and the secondary outcomes were clinical outcomes, including respiratory physiological parameters, incidence of barotrauma, ICU-free days, and ventilation-free days.

**Results:**

A total of 7 trials enrolling 1598 patients were finally included in this meta-analysis. The results revealed that the use of NMBAs in moderate-to-severe ARDS could significantly decrease the mortality truncated to day 28 (RR 0.74, 95% CI 0.56 to 0.98, *P* = 0.03) and day 90 (RR 0.77, 95% CI 0.60 to 0.99, *P* = 0.04). NMBA use could significantly decrease the incidence of barotrauma (RR 0.56, 95% CI 0.36 to 0.87, *P* = 0.009). No significant difference was observed in ICU-free days or ventilation-free days between the NMBA and control groups.

**Conclusion:**

The use of NMBAs could significantly decrease mortality in moderate-to-severe ARDS patients and decrease the incidence of barotrauma during mechanical ventilation. However, more large-scale randomized trials are needed to further validate the effect of NMBA use in ARDS.

## Background

Acute respiratory distress syndrome (ARDS) is defined as diffuse alveolar epithelial damage due to a dysregulated inflammatory response of intrapulmonary origin or to a systemic inflammatory process, resulting in refractory hypoxemia, reduced pulmonary compliance, and bilateral pulmonary infiltrates on chest imaging [1, 2]. ARDS represents approximately one-tenth of all intensive care unit (ICU) admittances, and its mortality surpasses 40% once it deteriorates to severe ARDS, which is a great challenge to ICU intensivists and a heavy burden to public health [3].

Over the past decades, only a few approaches have been proven to be effective in reducing mortality in ARDS patients, including a lung-protective ventilation strategy [4–6] and prone positioning [7, 8]. Neuromuscular blocking agents (NMBAs) have been the only pharmaceutical approach that is supposed to benefit patients with ARDS.

In ARDS, an excessive respiratory drive could lead to increased tidal volume, voluntary exhalation, and patient-ventilation asynchrony, which potentially worsen ventilator-induced lung injury (VILI) and increase mortality [9]. NMBAs prevent the patient-initiated generation of high volumes and active exhalation, facilitate patient-ventilator synchrony, provide protection from VILI, and ultimately reduce mortality in patients with ARDS [10–12]. In addition, NMBAs are also reported to directly alleviate pulmonary and systemic inflammatory progression [13]. In the updated guideline for ARDS management, NMBAs are recommended for a subgroup of moderate-to-severe ARDS patients for early use in 48 to 72 h [14]. However, NMBA use and inappropriate sedation are also associated with ICU-acquired weakness, delayed ventilation weaning, and prolonged hospital stay [15–18].

Previous meta-analyses have demonstrated a significant decrease in mortality in ARDS patients by NMBA use [19, 20]. However, in a recently published large-scale randomized trial in patients with moderate-to-severe ARDS, the early use of NMBAs in the first 48 h unexpectedly revealed no significant difference in mortality compared with usual care [21], which made NMBA use in ARDS further undetermined. Thus, it requires us for a quantitative analysis of the pooled results of up-to-date trials of NMBA use to validate the current effects of NMBAs in patients with moderate-to-severe ARDS. Furthermore, as the results between trials were inconsistent, we thought it might be necessary to investigate the reasons leading to the discrepancies in results for future study design and selection of patients that would potentially benefit from NMBA use in clinical practice.

## Methods

This work was performed according to the Preferred Reporting Items for Systematic Reviews and Meta-Analyses (PRISMA) statement [22, 23] (Additional File 1) and was registered in the International Prospective Register of Systematic Reviews (PROSPERO, CRD42019138416).

### Information sources

Two researchers independently searched several databases, including PubMed, EMBASE, Cochrane Central Register, and Web of Science up to June 1, 2019, and an updated search was conducted on November 14, 2019. When potentially relevant reviews or meta-analyses were found, a backward snowballing search was performed to obtain further studies.

### Search strategy

The following key words were used in the search: “neuromuscular blocking agents,” “neuromuscular blockers,” “acute respiratory distress syndrome,” “adult respiratory distress syndrome,” and “shock lung.” The full search strategy is included in Additional File 2**.**

### Eligibility criteria

Trials that met the following criteria were included in this meta-analysis: (1) study population of moderate-to-severe ARDS patients diagnosed according to the Berlin criteria [24] or American-European Consensus Conference (AECC) criteria for ARDS [25]; (2) early use of an NMBA of any category, with no restrictions on the duration or dosage of the NMBA; (3) data on mortality were reported; and (4) randomized control trial (RCT) study design. The exclusion criteria were as follows: (1) duplicates or overlapping populations, (2) in vitro or animal experiments, (3) pediatric or pregnant subjects, (4) no ARDS patients, and (5) lack of data on mortality.

### Study selection

Titles and abstracts were first reviewed separately by two researchers. When potentially relevant studies were found, the complete manuscripts were retrieved for further inspection. All the articles were reviewed, assessed, and selected by the two researchers independently with any disputes solved by consensus or consultation with a third researcher.

### Data items

The following information was extracted from the studies: (1) subject characteristics (including age, sex, onset and origin of ARDS), (2) interventions (including NMBA regimen, sedation strategy, and mechanical ventilation mode), and (3) outcome measurements. The primary outcome was mortality, and the secondary outcomes were clinical outcomes, including partial pressure of arterial oxygen and carbon dioxide (PaO_2_ and PaCO_2_), arterial pH, FiO_2_, PaO_2_ to FiO_2_ ratio (PaO_2_/FiO_2_), tidal volume, minute ventilation, peak inspiratory pressure (PIP), plateau pressure, respiratory rate, positive end-expiratory pressure (PEEP), driving pressure (DP), incidence of barotrauma, ICU-free days, and ventilation-free days.

The continuous variables were converted and are described as the mean with standard deviation (SD) if the median with interquartile range (IQR) or 95% confidence interval (CI) were reported. If the clinical variables were reported at baseline and after treatment, the alterations in the variables from baseline to posttreatment were calculated using the methods described in Additional File 3. DP was calculated by subtracting PEEP from plateau pressure, which was calculated whenever possible, using the methods provided in Additional File 3.

### Risk of bias assessment

Internal validity and risk of bias were assessed by two researchers separately following the Cochrane Collaboration’s protocols [26]. The risk of bias of the articles was evaluated as “yes”, “no,” or “unclear” after scrutinizing the procedures performed in the articles.

### Summary measures

Categorical variables are presented as proportions and were compared by risk ratios (RRs) with 95% CIs. Continuous variables are described as the mean ± SD and were compared by the mean difference (MD) or standard mean difference (SMD) according to the units of the variables.

### Statistical analysis

The data extracted from the articles were analyzed by Review Manager 5.3 (The Nordic Cochrane Centre, Cochrane Collaboration, Copenhagen). Mantel-Haenszel statistics were applied for categorical variable measurements, and an inverse variance model was used for continuous variables. A random-effects model was deployed for a better accommodation of heterogeneity. Cochrane *I*^2^ statistics were used to assess the statistical heterogeneity between studies, with *I*^2^ > 50% as high heterogeneity. Each study was sequentially removed, and the remaining dataset was reanalyzed for statistical significance or to detect favoring directions to evaluate the robustness of the results. Univariate meta-regression was used to explore the potential sources of heterogeneity. Post hoc sub-group analysis was conducted to analyze the effects of NMBA on sub-population of patients. Funnel plots were used to evaluate the publication bias of the studies by visual inspection.

Trial sequential analysis (TSA) was deployed to calculate the optimal information size [27] and was analyzed by Copenhagen Trial Unit’s Trial Sequential Analysis software (Copenhagen Trial Unit, Copenhagen). We estimated 36% mortality in the control arm and a reduction of mortality to 27% in the NMBA arm, adopted from the ACURASYS study [10], with 80% power and a two-sided alpha of 0.05. We inspected the Lan-DeMets sequential monitoring boundary and the current information size to determine whether the optimal information size was reached. A two-tailed *P* value less than 0.05 was considered statistically significant.

## Results

### Study selection and characteristics

**Fig. 1:**
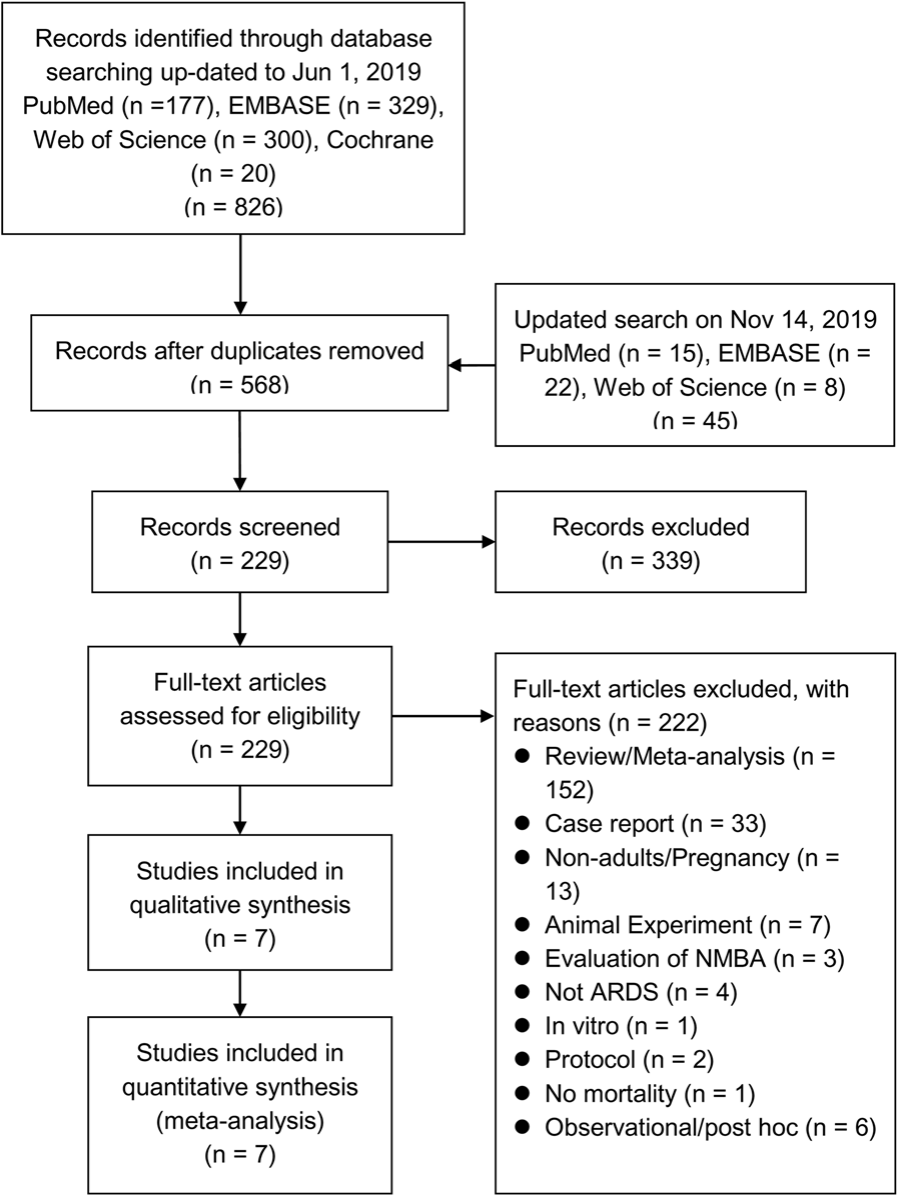
Flow chart of the search process and study selection

The comprehensive search yielded a total of 871 articles, and 7 randomized trials enrolling 1598 patients were finally included in this meta-analysis [10, 21, 28–32] (Fig. 1). Among the enrolled studies, three studies adopted the AECC criteria [10, 28, 29] and the remaining four used Berlin criteria [21, 30–32] for ARDS diagnosis. All studies used deep sedations with a Ramsay score of 6 in the control group, except for the ROSE study [21] and study by Rao et al. [32], in which light sedation with a Richmond Agitation-Sedation Scale (RASS) score of 0 to − 1 and a Ramsay score of 2 to 4 were used in the control arm respectively (Table 1). The detailed risk bias assessment of the trials is provided in Additional File 4.

**Table 1:** Baseline characteristics of the included trials

Study	Patients no. (sites)	Target patients	NMBA group	Control group	Ventilation strategy	Sedation target
Gainnier et al. [28]	56 (4)	AECC criteria for ARDS with PaO_2_/FiO_2_ ratio < 150 at a PEEP ≥ 5 cm H_2_O within 36 h	One bolus of 50 mg of cisatracurium infusion, followed by a continuous infusion at an initial rate of 5 μg/kg per min for 48 h	An infusion of saline at a rate of 4 mL/h	Assist-control mode for at least 48 h. Tidal volume 6–8 mL/kg of ideal body weight	Midazolam and sufentanil to obtain a Ramsay score of 6
Forel et al. [29]	36 (3)	AECC criteria for ARDS with PaO_2_/FiO_2_ ratio < 200 mmHg at a PEEP ≥ 5 cm H_2_O within 48 h of onset	One bolus of 50 mg of cisatracurium infusion, followed by a continuous infusion at an initial rate of 5 μg/kg per min for 48 h	An infusion of saline at a rate of 4 mL/h	Assist/control-volume with a tidal volume of 4–8 mL/kg predicted body weight and a plateau pressure of ≤ 30 cm H_2_O	Midazolam (3–30 mg/h) and sufentanil (10–150 μg/h) to obtain a Ramsay score of 6
Papazian et al. [10]	339 (12)	AECC criteria for ARDS with PaO_2_/FiO_2_ ratio < 150 mmHg at a PEEP ≥ 5 cm H_2_O within 48 h	One bolus infusion of 15 mg of cisatracurium, followed by a continuous infusion of 37.5 mg per hour for 48 h	An infusion of saline at the same rate as NMBA group	Volume assist-control mode, with a tidal volume of 6 to 8 mL per kilogram of predicted body weight	Obtain a Ramsay score of 6
Lyu et al. [30]	96 (1)	Moderate to severe ARDS with a PaO2/FiO2 less than 150 with PEEP at least 5 within the first 48 h	One bolus infusion of 0.1 mg/kg vecuronium, followed by a continuous infusion of 0.05 mg/kg per hour for 24 to 48 h	Usual care	Volume control mode, with a tidal volume of 4 to 8 mL per kilogram of predicted body weight	Sedation of midazolam and sufentanil
Rao et al. [32]	41 (1)	Berlin criteria for moderate-to-severe ARDS	Continuous infusion of 1 μg/kg per min of vecuronium	Usual care	Protective ventilation strategy, with a tidal volume of 6 mL per kilogram of ideal predicted body weight, with a plateau pressure ≤ 30 cm H2O	Midazolam and sufentanil or midazolam and fentanyl with a Ramsay score of 2 to 4
Guervilly et al. [31]	24 (2)	Moderate to severe ARDS with a PaO2/FiO2 less than 150 with PEEP at least 5 within the first 48 h	Cisatracurium besylate was given using a 3-mL rapid intravenous infusion of 15 mg followed by a continuous infusion of 37.5 mg/h	Usual care	Volume-assist control mode with a tidal volume of 6 mL/kg/IPBW (ideal predicted body weight)	Midazolam and sufentanil to achieve a Ramsay score of 6
Moss et al. [21]	1006 (48)	present for less than 48 h: PaO2:FiO2 of less than 150 mmHg with a PEEP of 8 cm or more of water	An intravenous bolus of 15 mg of cisatracurium, followed by a continuous infusion of 37.5 mg per hour for 48 h	An infusion of saline at the same rate as NMBA group	Protective ventilation strategy, with a tidal volume of 6 mL per kilogram of ideal predicted body weight, with a PEEP ≥ 8 cmH2O	A RASS score of 0 or − 1 in the control group

### Synthesis of results

#### Primary outcomes

**Fig. 2:**
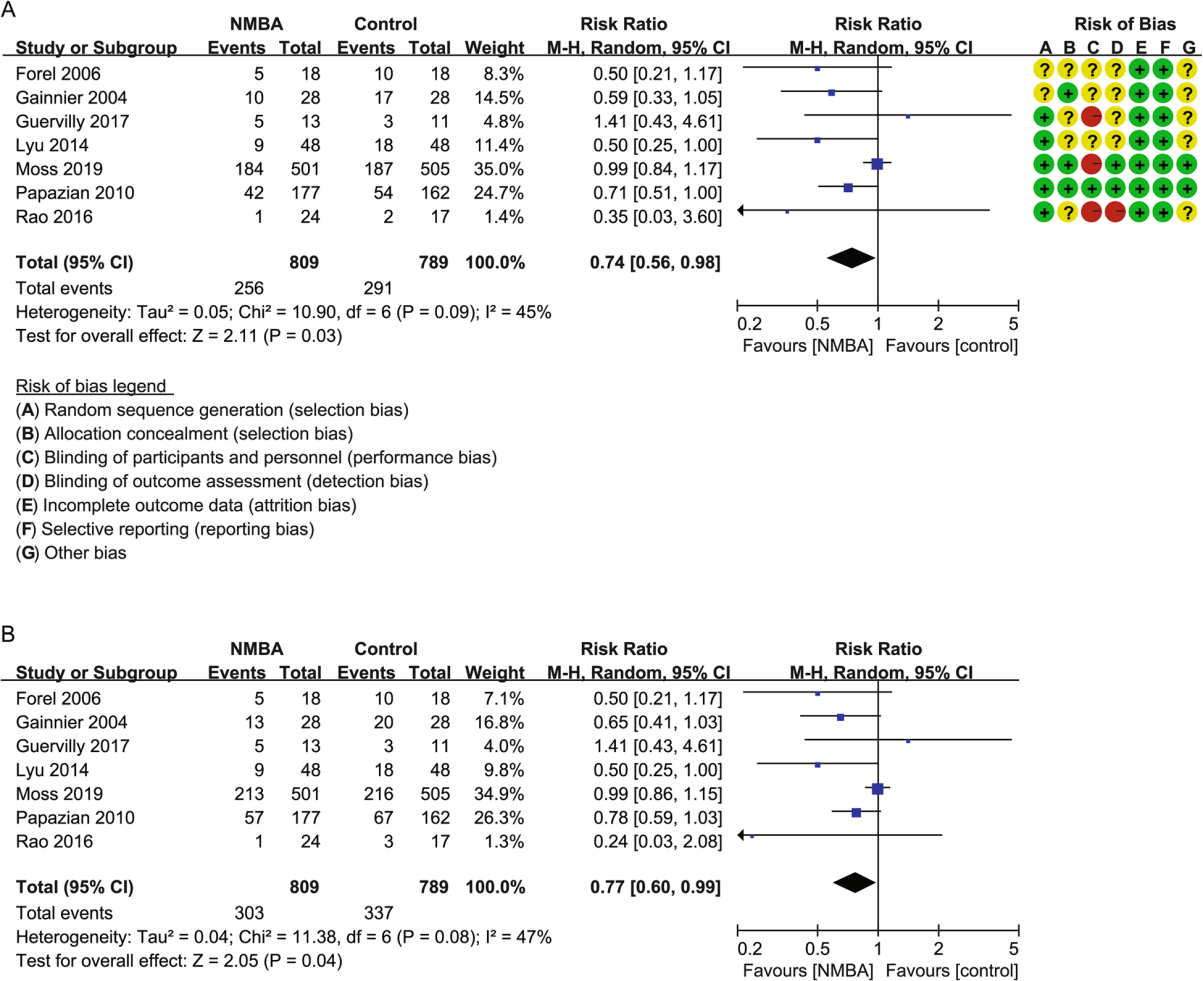
The effect of NMBAs on mortality truncated to day 28 (**a**) and day 90 (**b**) in moderate-to-severe ARDS patients. **a** The effect of NMBA on mortality truncated to day 28 in moderate-to-severe ARDS patients. **b** The effect of NMBA on mortality truncated to day 90 in moderate-to-severe ARDS patients

The data on mortality extracted from the trials were pooled and analyzed, and the results revealed a significant decrease in mortality in the NMBA group compared with the control group, with an RR of 0.74 (95% CI 0.56 to 0.98, *P* = 0.03), when the observation endpoint was truncated to day 28. The decrease in mortality in the NMBA group could still be observed, with an RR of 0.77 (95% CI 0.60 to 0.99, *P* = 0.04), when truncated to day 90 (Fig. 2).

#### Secondary outcomes

We compared the clinical outcomes between the NMBA and control groups and found that the use of NMBAs significantly increased the PaO_2_/FiO_2_ ratio change (mmHg, MD 8.97, 95% CI 0.66 to 17.28, *P* = 0.03); reduced FiO_2_ by approximately 4% (MD − 0.04, 95% CI − 0.09 to 0.00, *P* = 0.03); decreased the minute ventilation change (L/min, MD -0.51, 95% CI − 0.76 to − 0.07, *P* = 0.02); and decreased the PEEP level change (cmH_2_O, MD − 0.52, 95% CI − 1.01 to − 0.03, *P* = 0.04). The use of NMBAs also decreased the incidence of barotrauma in mechanical ventilation (RR 0.56, 95% CI 0.36 to 0.87, *P* = 0.009). We found a significant increase in DP in patients using NMBAs (cmH_2_O, MD 0.91, 95% CI 0.37 to 1.45, *P* = 0.001).

No significant differences could be observed in the other respiratory physiological parameters, ICU-free days (*P* = 0.74) or ventilation-free days (*P* = 0.19 when truncated to day 28, *P* = 0.09 when truncated to day 90) between the NMBA and control groups. The full list of comparisons of the clinical outcomes was provided in Additional File 5.

### Risk of bias and sensitivity analysis

We used both fixed-effects and random-effects models to retest the results, and we found no changes in favoring directions in either model. However, when each trial was sequentially omitted from the meta-analysis to analyze the individual effects of the trial on the overall results, the results showed that the significance was lost when some studies were omitted from the pooled analysis (Additional File 6). Visual inspection indicated asymmetry in the funnel plot, which indicated potential publication bias (Additional File 7).

### Meta-regression and subgroup analysis

We conducted a univariate meta-regression and found that the publishing year (*P* = 0.037), sample size (*P* = 0.036), and sedation strategy (deep vs. light sedation, *P* = 0.049) might associate with the heterogeneity between studies. Furthermore, the estimated improvement of PaO_2_ to FiO_2_ ratio 24 h after enrollment yielded a *P* value of 0.062. The full list of factors involved in the meta-regression was provided in Additional File 8. A post hoc sub-group analysis was deployed and found that patients diagnosed according to AECC criteria (RR 0.66, 95% CI 0.50 to 0.87, *P* = 0.003), patients with PaO2 to FiO2 ratio less than 150 mmHg at enrollment (RR 0.72, 95% CI 0.53 to 0.97, *P* = 0.03), and patients with deep sedation strategy (RR 0.66, 95% CI 0.51 to 0.84, *P* = 0.001) were likely to benefit from NMBA use (see Additional File 9).

### Trial sequential analysis

**Fig. 3:**
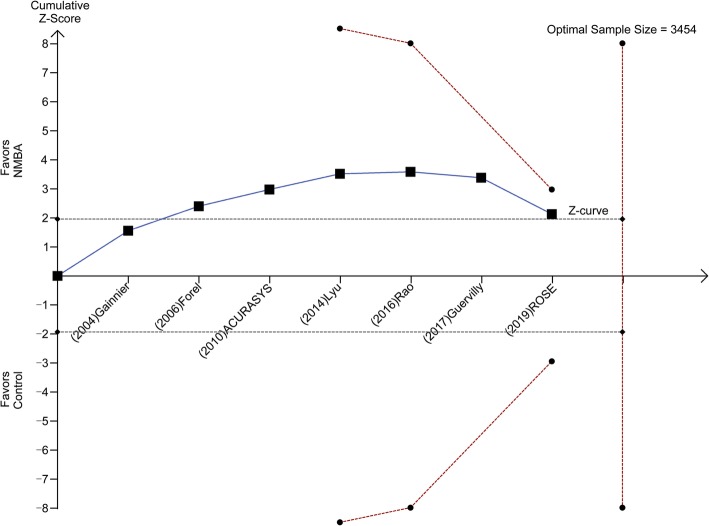
Trial sequential analysis revealing the optimal sample size for detecting the plausible effect of NMBA use

Trial sequential analysis was performed in the study, and the results indicated that the current information size did not cross the Lan-DeMets sequential monitoring boundary constructed by the optimal information size, indicating insufficient sample size in investigating the mortality truncated to day 28. An optimal sample size of 3454 patients was estimated, which was supposed to reach the plausible endpoint (Fig. 3).

## Discussion

In the present meta-analysis, we pooled the results from 7 trials enrolling 1598 patients to validate the use of NMBAs in patients with moderate-to-severe ARDS and found that the early use of NMBAs in the first 48 h could significantly reduce mortality truncated to day 28 (RR 0.74, 95% CI 0.56 to 0.98, *P* = 0.03) and to day 90 (RR 0.77, 95% CI 0.60 to 0.99, *P* = 0.04). The use of NMBAs was associated with improved respiratory parameters, including increased PaO_2_/FiO_2_ ratio and reduced FiO_2_, minute ventilation, PEEP level, and incidence of barotrauma during mechanical ventilation (RR 0.56, 95% CI 0.36 to 0.87, *P* = 0.009). We also found an increased measurement of DP after NMBA use (cmH_2_O, MD 0.91, 95% CI 0.37 to 1.45, *P* = 0.001).

Our results were consistent with previous findings that the use of NMBAs could significantly reduce mortality in patients with moderate-to-severe ARDS [19, 20]. NMBA use was associated with facilitated patient-ventilator synchrony, which potentially reduces the incidence of VILI and barotrauma and ultimately improves outcomes in ARDS patients.

We found a significant increase in DP in the NMBA group compared with the control group (cmH_2_O, MD 0.91, 95% CI 0.36 to 1.45, *P* = 0.001). We speculated that this finding may be due to the existence of spontaneous breath in the control group, which led to the inaccurate measurement of DP.

However, sensitivity analysis suggested that our result was not robust and Cochrane *I*^2^ statistics indicated certain heterogeneity between studies (*I*^2^ = 45%).

Sedation strategy might be one of the sources of heterogeneity. Sub-group analysis suggested that ARDS patients underwent deep sedation was more likely to benefit from NMBA use (RR 0.66, 95% CI 0.51 to 0.84, *P* = 0.001). We thought this could be partially explained by the effects of reverse triggering, defined as a diaphragm contraction triggered by mechanical ventilation, which could lead to breath stacking, VILI, and barotrauma [33]. Reverse triggering was paradoxically seen in ARDS patients with deep sedation and could be resolved by NMBA use [34]. It was possible that in the trials included in the present meta-analysis, the patients in the control group with deep sedation would have reverse triggering and thus worse outcomes, and the use of NMBAs could resolve the deleterious effect of reverse triggering, while in studies in which light sedation was used in the control group, reverse triggering was mitigated, which might have masked the effects of NMBA use [35, 36].

We saw a tendency of statistical significance by meta-regression that the resolve of PaO_2_/FiO_2_ ratio on the second day after enrollment was likely to contribute to the source of heterogeneity (*P* = 0.062). We speculated that the ARDS patients who had a greater improvement in PaO_2_/FiO_2_ ratio were probably not as severe or at least not as refractory reversible, which suggested that the use of NMBAs might be beneficial in patients with extremely refractory and severe ARDS.

We also noticed a significant effect of NMBA use in a sub-group of ARDS patients with a PaO_2_/FiO_2_ ratio less than or equal to 150 mmHg at enrollment (RR 0.72, 95% CI 0.53 to 0.97, *P* = 0.03). However, as the disparity of the number of patients in each sub-group was great, this result should be interpreted with much caution. Furthermore, meta-regression suggested that the year in which the study was published also introduced heterogeneity (*P* = 0.037) and we found NMBA use was effective in the ACEE definition for ARDS (RR 0.66, 95% CI 0.50 to 0.87, *P* = 0.003). However, we did not regard the definition for ARDS as a confounding factor and we would rather believe this could probably due to the evolvement of ARDS management, which seemed to make NMBA use less important. NMBA was not a “magic bullet” and should be a part of a lung-protective strategy.

The current results may not be conclusive. We then conducted TSA; the results indicated that more patients were needed to validate the use of NMBAs in ARDS, with an optimal sample size of 3454 patients.

Our study had several limitations. Some trials included in the meta-analysis were of limited sample size, which was likely to bring bias to the results, and only two large-scale randomized trials were included in our meta-analysis. More trials are needed to further validate the effects of NMBAs in ARDS. We used a mathematical imputation method to calculate the variables with the after-before alterations of certain parameters, which would potentially bring bias to the results and the interpretations. Some of the subgroup analyses included only two studies, which could potentially bring bias to the results and should be interpreted cautiously.

## Conclusion

Early use of NMBAs could reduce mortality and decrease the incidence of barotrauma during mechanical ventilation in patients with moderate-to-severe ARDS. However, more RCTs are needed to further validate the effects of NMBAs in ARDS.

## Supplementary information

**Additional file 1.** Checklist for systematic reviews applied on this manuscript according to the Preferred Reporting Items for Systematic reviews and Meta-Analysis (PRISMA).

**Additional file 2.** Full list of search strategy for PubMed.

**Additional file 3.** Data transformation and missing data imputations.

**Additional file 4.** Risk of bias assessment of the included trials.

**Additional file 5.** Secondary outcome analysis, including PaO2 to FiO2 ratio, PaO2, arterial pH, FiO2, PIP, Pplat, tidal volume, PEEP, minute ventilation, DP, MRC score, respiratory rate, barotrauma, ICU-free days and ventilation-free days.

**Additional file 6.** Sensitivity analysis.

**Additional file 7.** Publication bias by funnel plot.

**Additional file 8.** Univariate meta-regression analysis. Following factors were used for meta-regression, publishing year, sample size, language (English or Chinese), ARDS definition (AECC or Berlin criteria), estimated average PaO2 to FiO2 ratio of ARDS patients at enrollment, estimated average improvement of PaO2 to FiO2 ratio 24 hours after enrollment, quality of the study, percentage of patients with intra-pulmonary etiology, sedation strategy (light or deep).

**Additional file 9.** Sub-group analysis. Patients were divided by ARDS definition (AECC or Berlin), initial PEEP setting (<= 10 mmHg vs > 10mmHg), PaO2 to FiO2 ratio at enrollment (<= 150mmHg vs > 150mmHg) and sedation strategy (light vs deep sedation).

## Data Availability

The datasets used and/or analyzed during the current study are available from the corresponding author upon reasonable request.

## Abbreviations

AECC: American-European Consensus Conference
ARDS: Acute respiratory distress syndrome
CI: Confidence interval
DP: Driving pressure
ICU: Intensive care unit
IQR: Interquartile range
MD: Mean difference
NMBA: Neuromuscular blocking agent
OR: Odds ratio
PEEP: Positive end-expiratory pressure
PIP: Peak inspiratory pressure
PROSPERO: International Prospective Register of Systemic Reviews
RASS: Richmond Agitation-Sedation Scale
RCT: Randomized control trial
SD: Standard deviation
SMD: Standard mean difference
TSA: Trial sequential analysis
