# Supplementary material for: Validation of neuromuscular blocking agent use in acute respiratory distress syndrome: a meta-analysis of randomized trials
Source: Crit Care. 2020 Feb 17;24:54. doi: 10.1186/s13054-020-2765-2 (PMC7027110; doi:10.1186/s13054-020-2765-2)
Supplement: Supplementary file 2 — Additional file 2. Full list of search strategy for PubMed. [file 13054_2020_2765_MOESM2_ESM.docx]

# Search Strategy for PubMed

#14 Add Search ((((((((((Respiratory Distress Syndrome, Adult) OR Shock Lung) OR Lung, Shock) OR ARDS, Human) OR ARDSs, Human) OR Human ARDS) OR Respiratory Distress Syndrome, Acute) OR Acute Respiratory Distress Syndrome) OR Adult Respiratory Distress Syndrome)) AND (((((Neuromuscular Blocking Agents) OR Agents, Neuromuscular Blocking) OR Blocking Agents, Neuromuscular) OR Neuromuscular Blockers) OR Blockers, Neuromuscular) 177

#13 Add Search ((((((((Respiratory Distress Syndrome, Adult) OR Shock Lung) OR Lung, Shock) OR ARDS, Human) OR ARDSs, Human) OR Human ARDS) OR Respiratory Distress Syndrome, Acute) OR Acute Respiratory Distress Syndrome) OR Adult Respiratory Distress Syndrome 37662

#12 Add Search ((((Neuromuscular Blocking Agents) OR Agents, Neuromuscular Blocking) OR Blocking Agents, Neuromuscular) OR Neuromuscular Blockers) OR Blockers, Neuromuscular 27430
