# Supplementary material for: Validation of neuromuscular blocking agent use in acute respiratory distress syndrome: a meta-analysis of randomized trials
Source: Crit Care. 2020 Feb 17;24:54. doi: 10.1186/s13054-020-2765-2 (PMC7027110; doi:10.1186/s13054-020-2765-2)
Supplement: Supplementary file 3 — Additional file 3. Data transformation and missing data imputations. [file 13054_2020_2765_MOESM3_ESM.docx]

# Data transformation and missing data imputations

## *Standard deviation calculated from interquartile range*

The median is regarded as mean, and SD is calculated from the following method,

$$SD=\frac{Q3-Q1}{1.35}$$

## *Missing data of range change imputated from end-point and baseline*

The mean (mean_range_) and SD (SD_range_) of the range changes are calculated by the following formulas:

$${mean}_{range}={mean}_{end-point}-{mean}_{baseline}$$

$${SD}_{range}=\sqrt{{SD}_{baseline}^{2}+{SD}_{end-point}^{2}-2\times R\times{SD}_{baseline}\times{SD}_{end-point}}$$

Within which, *R* is called correlation coefficient and is regarded as 0.4 or 0.5 during the calculation.

## *Driving pressure imputated from plateau pressure (Pplat) and PEEP*

*The driving pressure (DP) and relevant SD (SD_DP_)are calculated by the following formulas:*

$$DP=Pplat-PEEP$$

$${SD}_{DP}=\sqrt{{SD}_{Pplat}^{2}+{SD}_{PEEP}^{2}-2\times R\times{SD}_{Pplat}\times{SD}_{PEEP}}$$

Within which, *R* is called correlation coefficient and is regarded as 0.4 or 0.5 during the calculation.

*Abbreviations: IQR inter-quartile range, SD standard deviation*
