# Supplementary material for: Validation of neuromuscular blocking agent use in acute respiratory distress syndrome: a meta-analysis of randomized trials
Source: Crit Care. 2020 Feb 17;24:54. doi: 10.1186/s13054-020-2765-2 (PMC7027110; doi:10.1186/s13054-020-2765-2)
Supplement: Supplementary file 4 — Additional file 4. Risk of bias assessment of the included trials. [file 13054_2020_2765_MOESM4_ESM.docx]

**Risk of bias assessment of the included trials**

| Gainnier 2004 | | |
| --- | --- | --- |
| Bias | **Authors' judgement** | **Support for judgement** |
| Random sequence generation  (selection bias) | **UNCLEAR** | **Quote** *“Randomization was stratified by intensive care unit, with equal numbers of patients in each treatment numbers of patients in each treatment group in variable-sized blocks” (P114)*  **Comment** The randomization method was not elucidated. |
| Allocation concealment  (selection bias) | **LOW** | **Quote** *“All members of the study team and clinical staffs were masked as to the study group assignment sequence and block size. Indeed, a sheet masked the infusion pump.” (P114)* |
| Blinding of participants and personnel  (performance bias) | **UNCLEAR** | **Quote** *“The investigators were blinded vis-à-vis the group assignment…The nurse in charge of the patient was responsible for the assessment of neuromuscular blockade and NMBA delivery…the cisatracurium infusion rate was adapted by the nonblinded nurse in charge of the patient according to a standardized protocol…” (P114)*  **Comment** The attending nurse was unblinded to the patient management, the nurse seemed not to participate in the data collection, however, the authors did not elucidate the roll the nurse played explicitly. |
| Blinding of outcome assessment  (detection bias) | **UNCLEAR** | **Quote** *“The investigators were blinded vis-à-vis the group assignment” (P114)*  **Comment** The authors did not elucidate who should collect and analyze data, considering the attending nurse was unblinded to the patients. |
| Incomplete outcome data  (attrition bias) | **LOW** | **Comment** All the data was reported |
| Selective reporting  (reporting bias) | **LOW** | **Comment** All the outcomes were reported |
| Other bias | **UNCLEAR** | **Comment** sample size was relatively small |

| Forel 2006 | | |
| --- | --- | --- |
| Bias | **Authors' judgement** | **Support for judgement** |
| Random sequence generation  (selection bias) | **UNCLEAR** | **Quote** *“Randomization was stratified by intensive care unit, with equal numbers of patients in each treatment group in variable-sized blocks” (P2750)*  **Comment** The randomization method was not elucidated. |
| Allocation concealment  (selection bias) | **UNCLEAR** | **Quote** *“The investigators were blinded vis-à-vis the group assignment.” (P2750)*  **Comment** The method of allocation concealment was not tell. |
| Blinding of participants and personnel  (performance bias) | **UNCLEAR** | **Quite** *“The nurse in charge of the patient was responsible for NMBA delivery and the assessment of neuromuscular blockade…The cisatracurium infusion rate was adapted by the nonblinded nurse…”*  **Comment** The attending nurse was unblinded to the patients. It was acceptable if only the attending nurse was unblinded, and the other investigators were still blinded to the assignment and management of the patients. However, the authors did not tell. |
| Blinding of outcome assessment  (detection bias) | **UNCLEAR** | **Comment** The authors did not tell who should collect and analyze the data, although theoretically the investigators blinded to the patients would collect the data. |
| Incomplete outcome data  (attrition bias) | **LOW** | **Comment** No outcome data was missing. |
| Selective reporting  (reporting bias) | **LOW** | **Comment** All the outcomes were reported. |
| Other bias | **UNCLEAR** | **Comment** Sample size relatively small. |

| Papazian 2010 | | |
| --- | --- | --- |
| Bias | **Authors' judgement** | **Support for judgement** |
| Random sequence generation  (selection bias) | **LOW** | **Quote** *“The trial was monitored by an independent data and safety monitoring board. Randomization and blinding regarding the study-group assignments were performed according to Consolidated Standards for the Reporting of Trials (CONSORT) guidelines…” (P1108)*  *“Computer-generated random-number tables prepared by statisticians were used to assign patients in blocks of 4 to either NMBA or placebo.” (Appendix)* |
| Allocation concealment  (selection bias) | **LOW** | **Quote** *“At each center, designated investigators enrolled the patients and called a centralized telephone system to ensure blind allocation of consecutively numbered boxes containing placebo or cisatracurium besylate” (Appendix)* |
| Blinding of participants and personnel  (performance bias) | **LOW** | **Quote** *“Cisatracurium besylate (150-mg formulation GlaxoSmithKline) and placebo were prepared in identical separate 30-ml vials for intravenous infusion…” (P1108)*  *“An open-label, rapid, intravenous injection of 20 mg of cisatracurium was allowed in both groups if the end-inspiratory plateau pressure remained greater than 32 cm of water…” (P1109)*  **Comment** The open-label injection of cisatracurium in both groups to decrease the plateau pressure could eliminate the imbalance between the NMBA and control group, which we thought might be sufficient in blindness. |
| Blinding of outcome assessment  (detection bias) | **LOW** | **Quote** *“Patients, healthcare provider, evaluators, monitors, and data analysts were also blinded to the study treatment”* |
| Incomplete outcome data  (attrition bias) | **LOW** | **Comment** All the missing data was reported and explained. |
| Selective reporting  (reporting bias) | **LOW** | **Comment** All outcomes were reported. |
| Other bias | **LOW** | **Commen**t None. |

| Lyu 2014 | | |
| --- | --- | --- |
| Bias | **Authors' judgement** | **Support for judgement** |
| Random sequence generation  (selection bias) | **LOW** | **Quote** *“…各组再按随机数字表法分为治疗组与对照组…” (“…each group was divided as treatment group and control group by random number table assignment…”)* |
| Allocation concealment  (selection bias) | **UNCLEAR** | **Comment** Methods for allocation concealment not mentioned in the main text |
| Blinding of participants and personnel  (performance bias) | **UNCLEAR** | **Comment** Methods for blinding of participants and personnel not mentioned |
| Blinding of outcome assessment  (detection bias) | **UNCLEAR** | **Comment** Methods for outcome assessment not mentioned |
| Incomplete outcome data  (attrition bias) | **LOW** | **Comment** All patient data reported |
| Selective reporting  (reporting bias) | **LOW** | **Comment** All outcomes reported |
| Other bias | **UNCLEAR** | **Comment** Sample size relatively small |

| Guervilly 2017 | | |
| --- | --- | --- |
| Bias | **Authors' judgement** | **Support for judgement** |
| Random sequence generation  (selection bias) | **LOW** | **Quote** *“…computer-generated random-number table stratified by center and prepared by statisticians to assign patients in blocks of 4 to receive or not a 48-h infusion of cisatracurium besylate…”* |
| Allocation concealment  (selection bias) | **UNCLEAR** | **Quote** *“…computer-generated random-number table stratified by center and prepared by statisticians to assign patients in blocks of 4 to receive or not a 48-h infusion of cisatracurium besylate…”*  **Comment** However, the method of concealment was not revealed in the main text. |
| Blinding of participants and personnel  (performance bias) | **HIGH** | **Comment** The method of blinding was not mentioned in the main text; however, we don’t think the blinding was well executed according to the author’s protocols. |
| Blinding of outcome assessment  (detection bias) | **UNCLEAR** | **Comment** The blinding of outcomes was not mentioned in the main text. |
| Incomplete outcome data  (attrition bias) | **LOW** | **Comment** There was no missing data in the outcomes |
| Selective reporting  (reporting bias) | **LOW** | **Comment** All outcomes reported |
| Other bias | **UNCLEAR** | **Comment** Sample size relatively small |

| Rao 2016 | | |
| --- | --- | --- |
| Bias | **Authors' judgement** | **Support for judgement** |
| Random sequence generation  (selection bias) | **LOW** | **Quote** *“…central net-work randomization system was used for random concealment…patients were allocated according to random number table …”* |
| Allocation concealment  (selection bias) | **UNCLEAR** | **Quote** *“…central net-work randomization system was used for random concealment…patients were allocated according to random number table …”*  **Comment** However, the method of concealment was not revealed in the main text. |
| Blinding of participants and personnel  (performance bias) | **HIGH** | **Quote** *“…prospective single-blinded randomised control trial…”*  **Comment** This was a single blinded trial. |
| Blinding of outcome assessment  (detection bias) | **HIGH** | **Comment** Since this was a single blinded trial, we thought it was not blinded to the stasticians. |
| Incomplete outcome data  (attrition bias) | **LOW** | **Comment** There was no missing data in the outcomes |
| Selective reporting  (reporting bias) | **LOW** | **Comment** All outcomes reported |
| Other bias | **UNCLEAR** | **Comment** Sample size relatively small |

| Moss 2019 | | |
| --- | --- | --- |
| Bias | **Authors' judgement** | **Support for judgement** |
| Random sequence generation  (selection bias) | **LOW** | **Quote** *“…using a computer generated, permuted block design, and stratification by institution, The CCC and protocol leaders provide continuous access for randomization backup”* |
| Allocation concealment  (selection bias) | **LOW** | **Quote** *“…via an automated centralized assignment system. Only after enrollment does the system assign a study arm…”* |
| Blinding of participants and personnel  (performance bias) | **HIGH** | **Quote** *“…chose to not blind cisatracurium administration…”* |
| Blinding of outcome assessment  (detection bias) | **LOW** | **Quote** *“We restrict access to unblinded data to designated study statisticians and oversight committees…”* |
| Incomplete outcome data  (attrition bias) | **LOW** | **Comment** All missing data reported |
| Selective reporting  (reporting bias) | **LOW** | **Comment** All outcomes reported |
| Other bias | **LOW** | **Comment** None. |
