# Supplementary figures and images for: Validation of neuromuscular blocking agent use in acute respiratory distress syndrome: a meta-analysis of randomized trials
Source: Crit Care. 2020 Feb 17;24:54. doi: 10.1186/s13054-020-2765-2 (PMC7027110; doi:10.1186/s13054-020-2765-2)

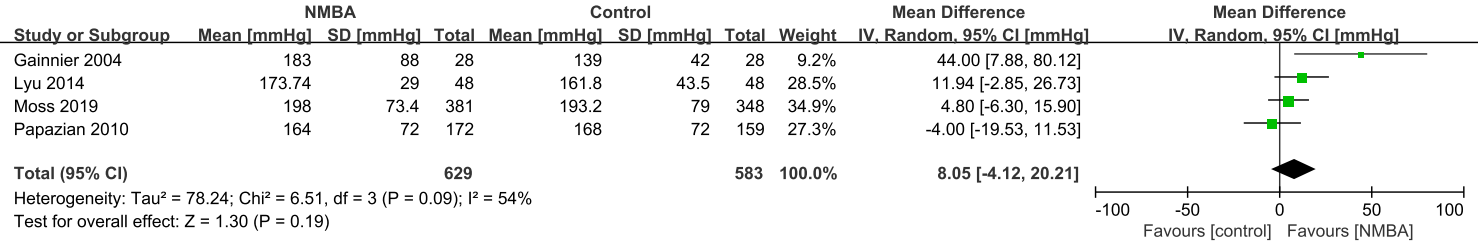

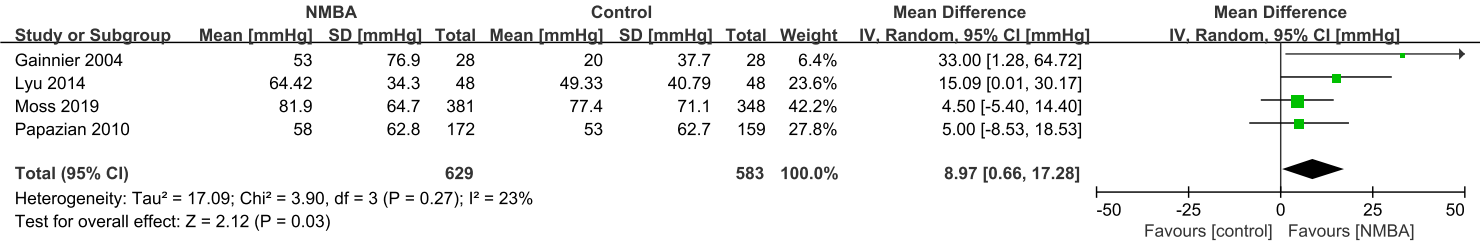

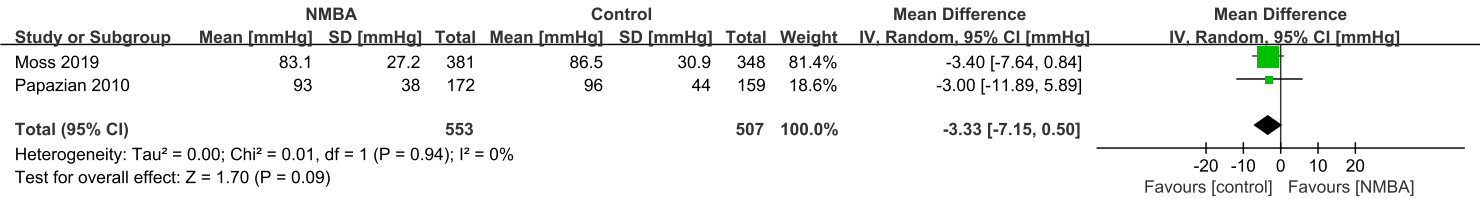

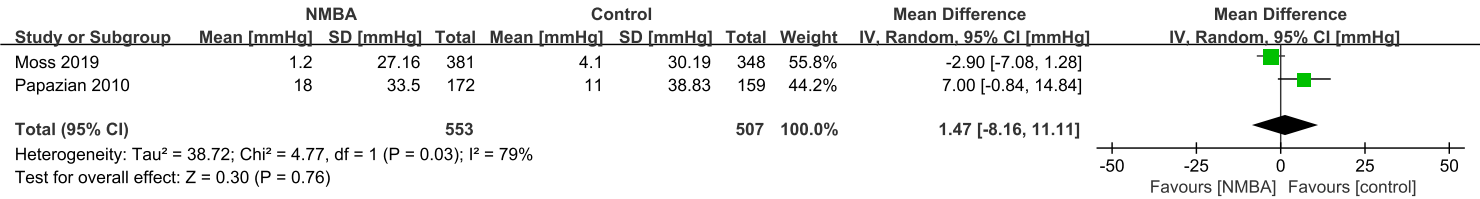

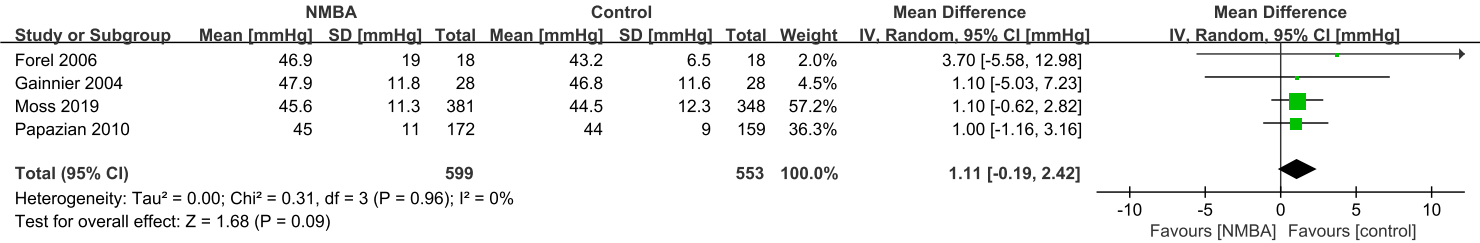

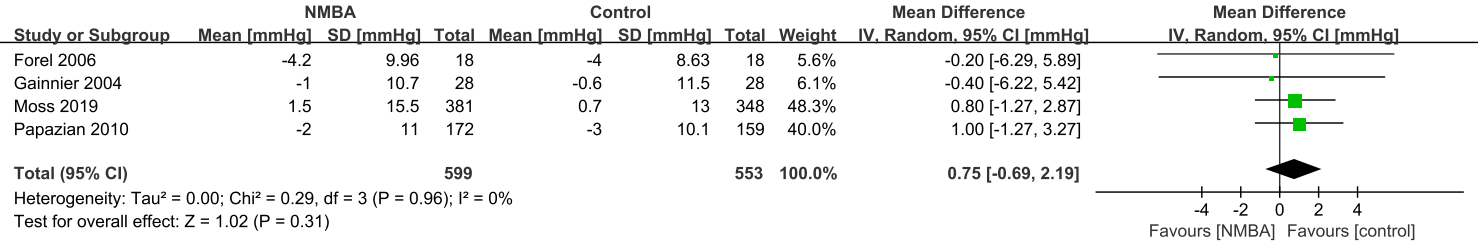

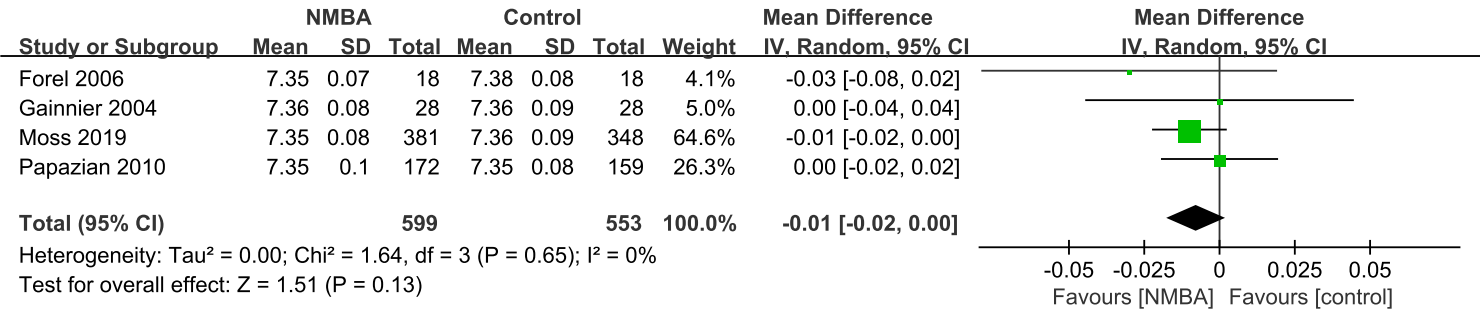

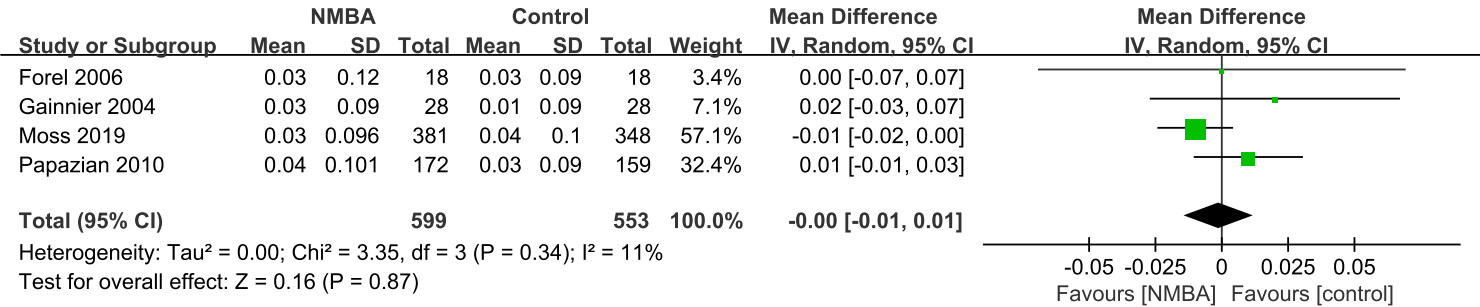

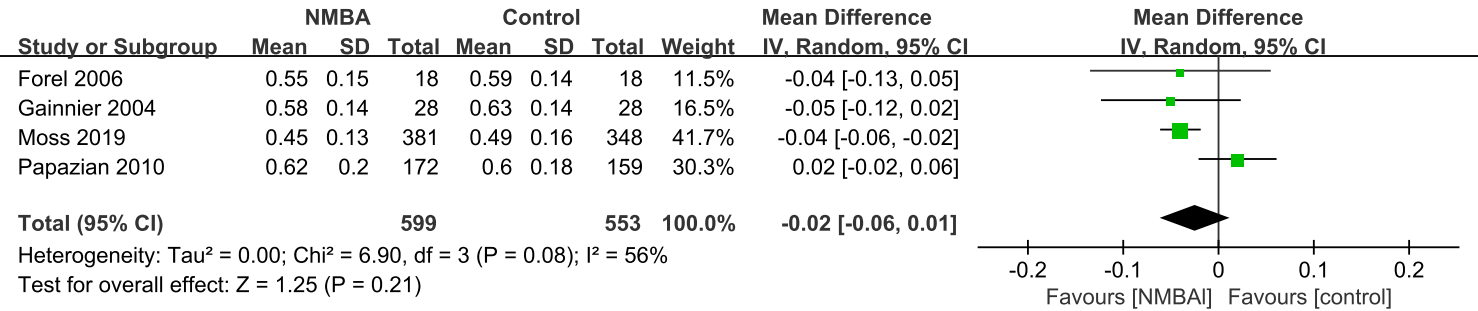

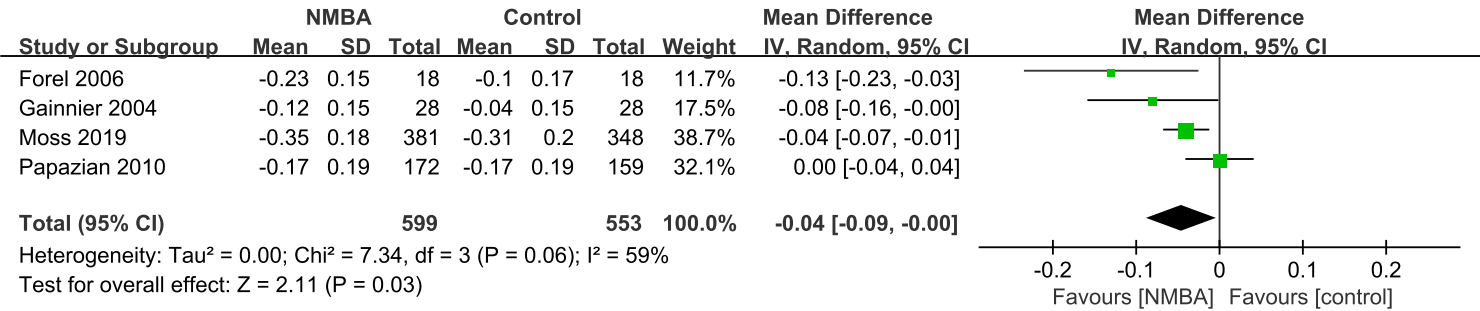

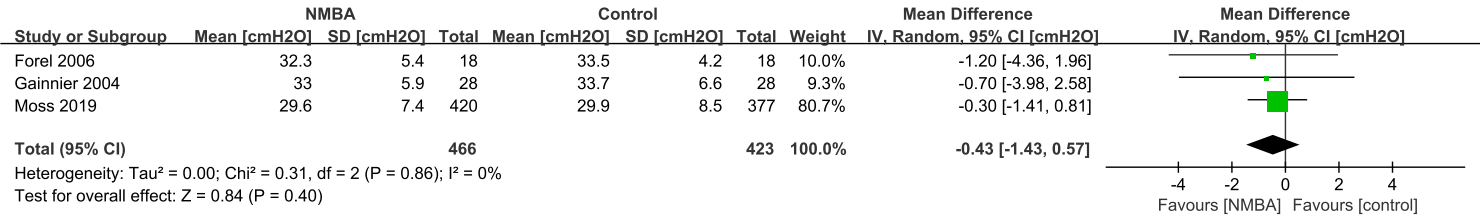

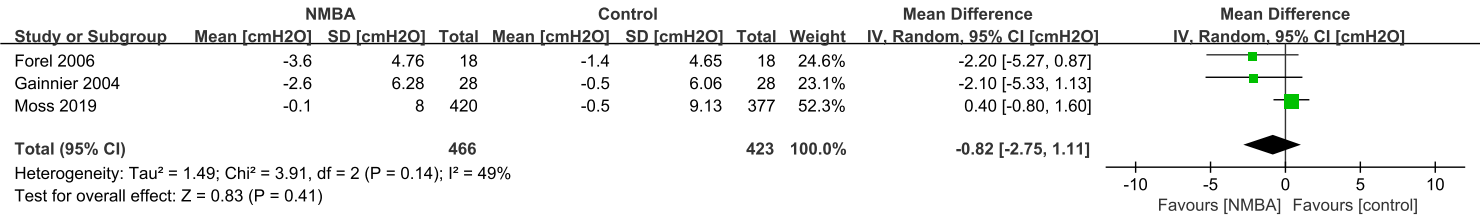

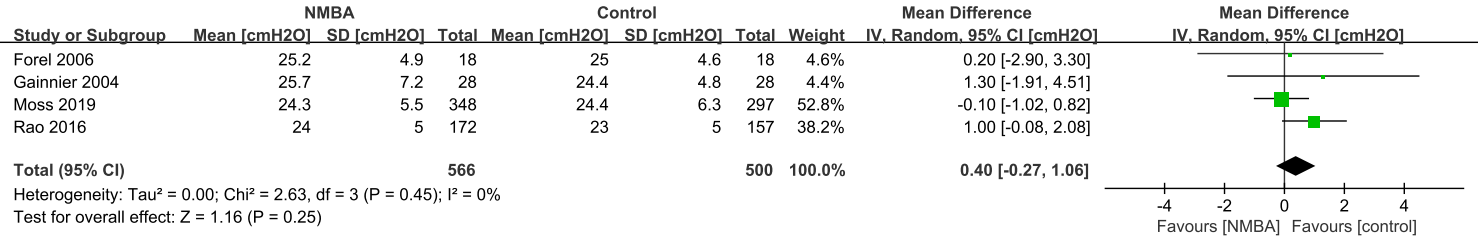

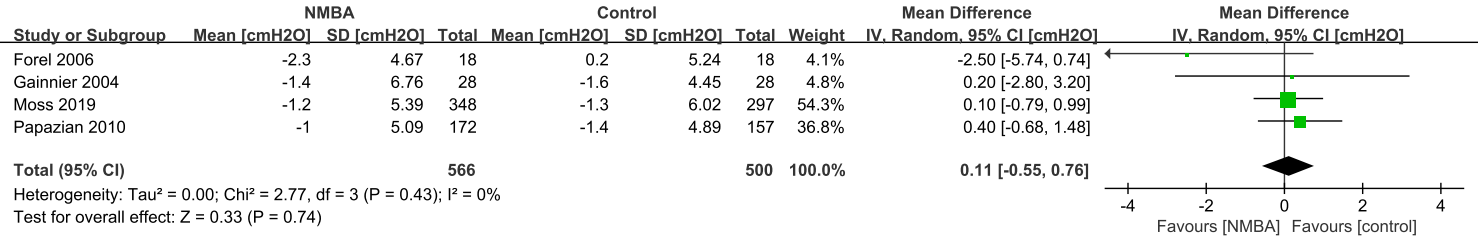

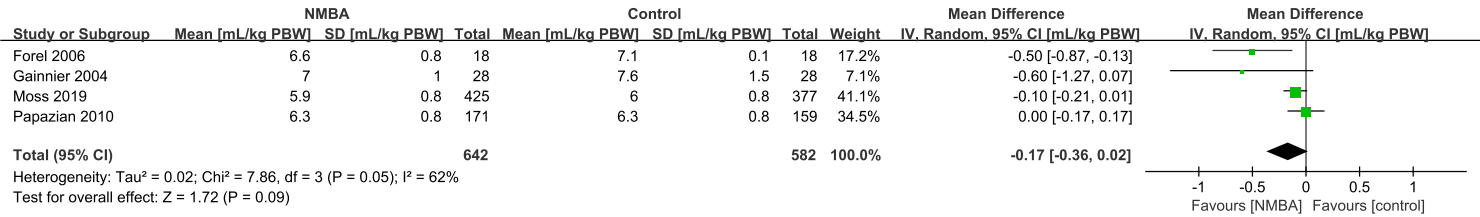

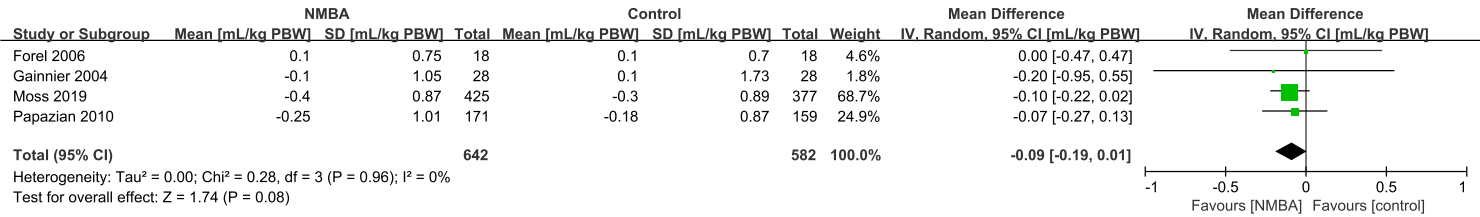

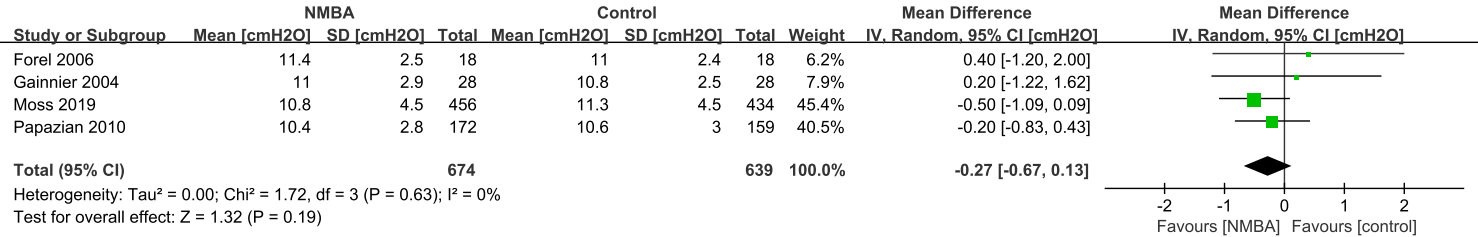

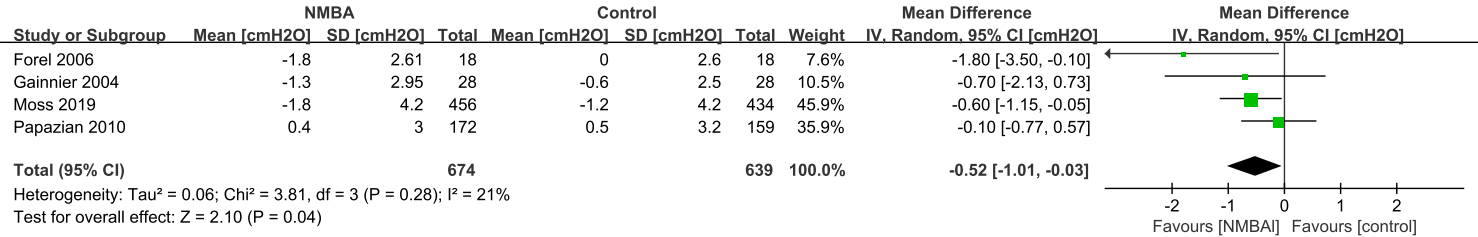

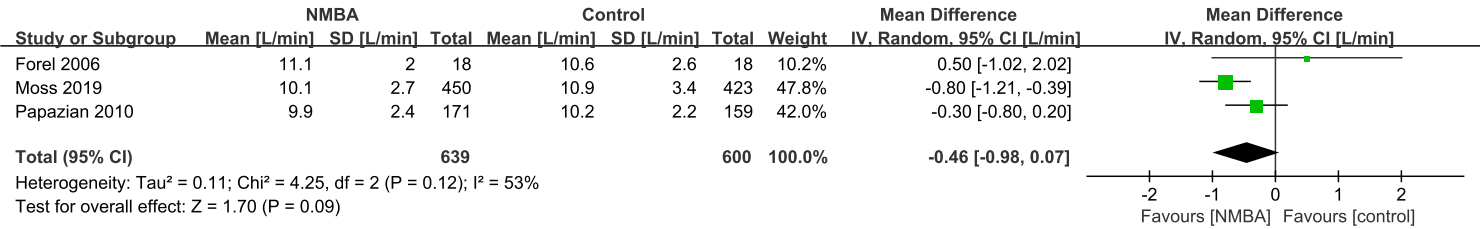

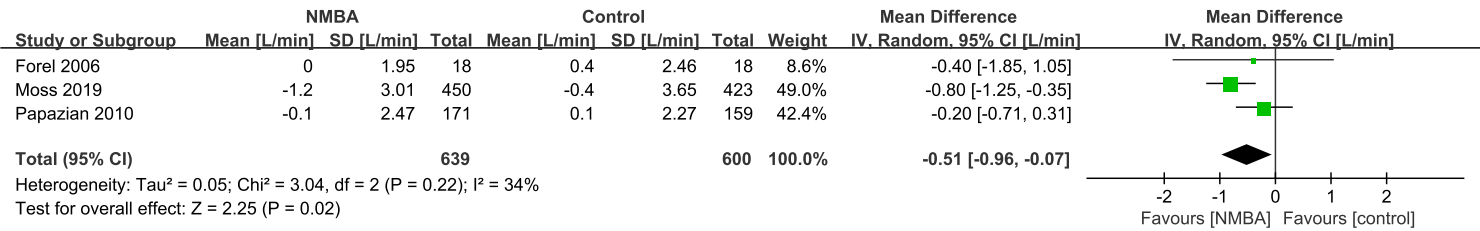

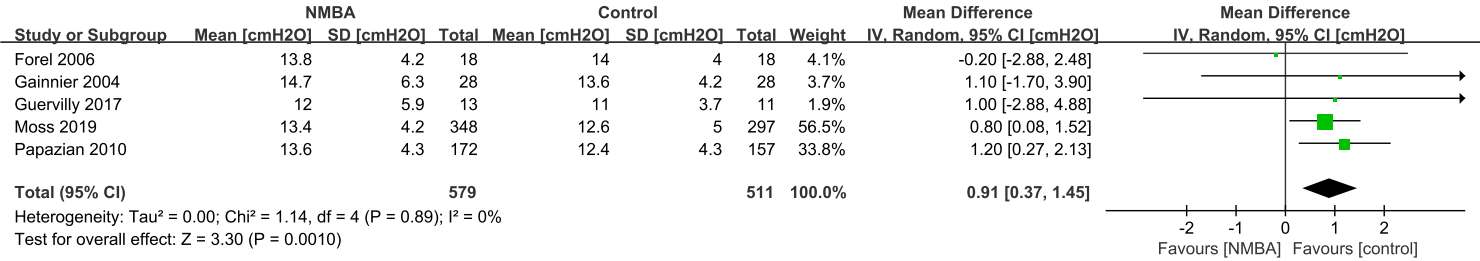

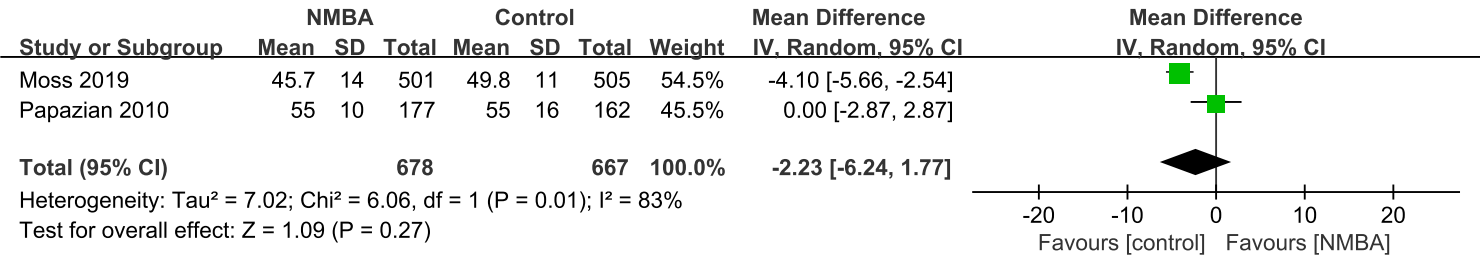

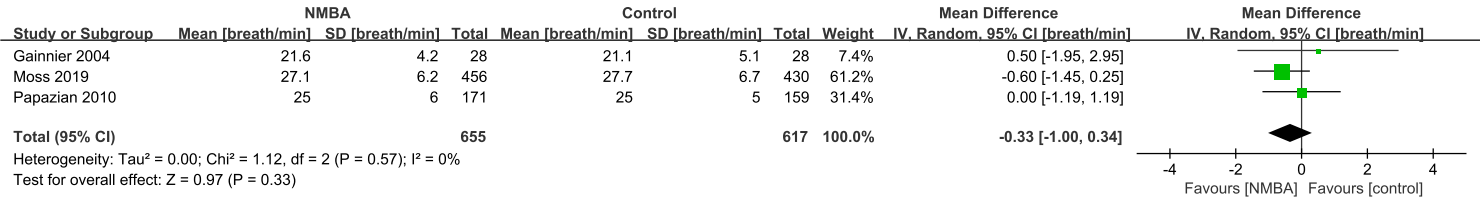

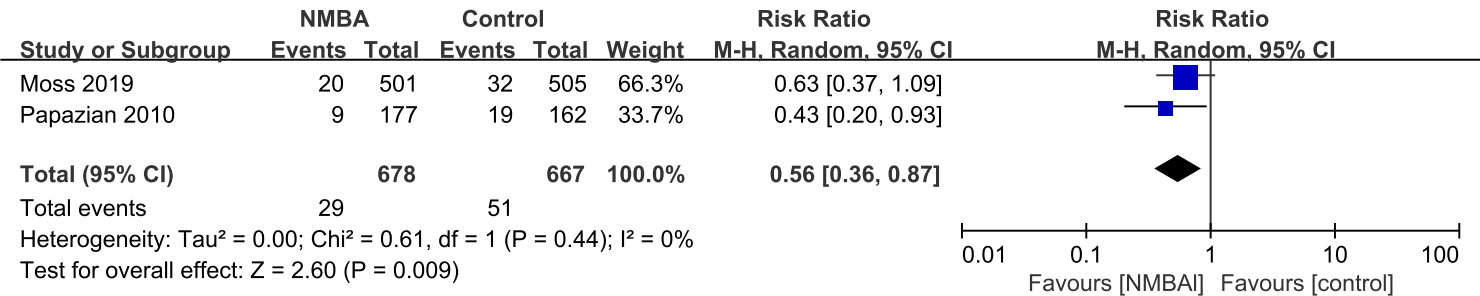

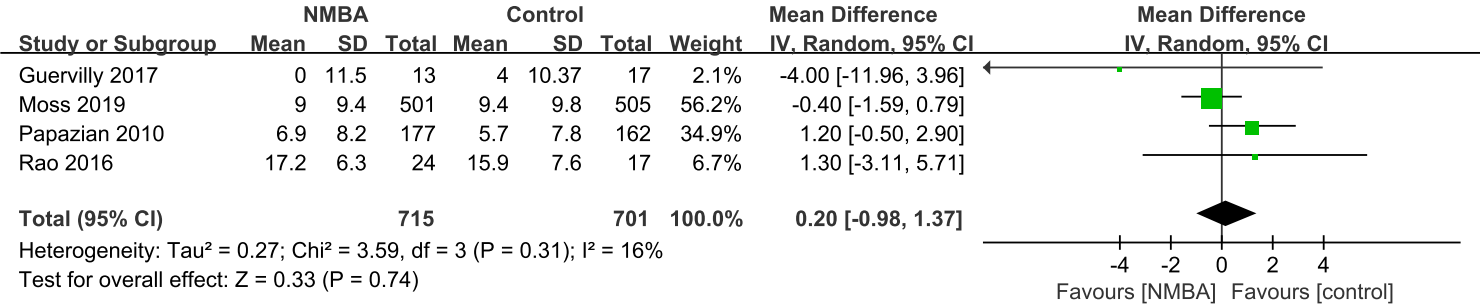

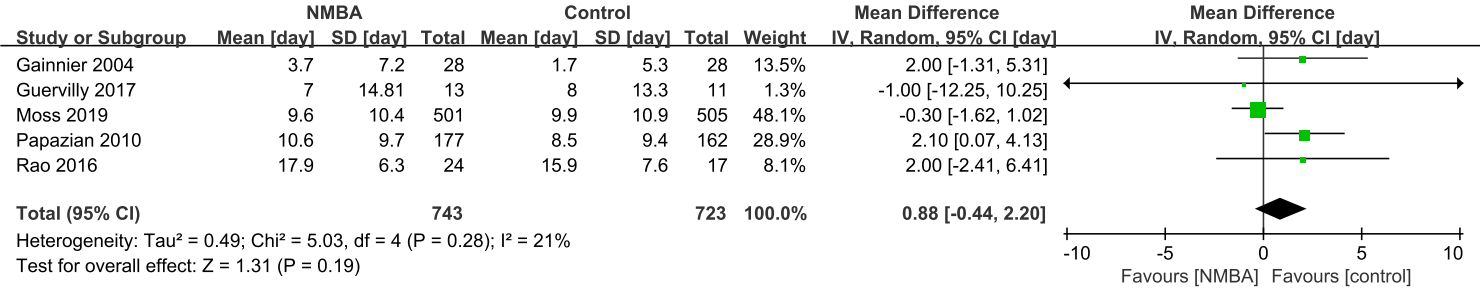

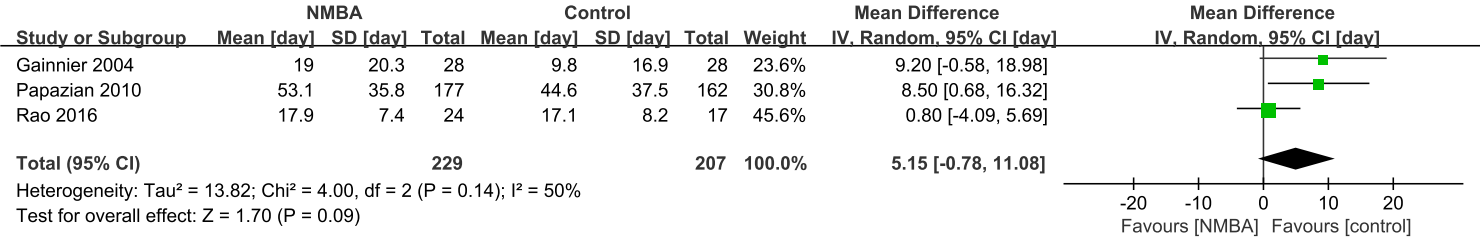

Supplement: Supplementary file 5 — Additional file 5. Secondary outcome analysis, including PaO2 to FiO2 ratio, PaO2, arterial pH, FiO2, PIP, Pplat, tidal volume, PEEP, minute ventilation, DP, MRC score, respiratory rate, barotrauma, ICU-free days and ventilation-free days. [file 13054_2020_2765_MOESM5_ESM.pdf]

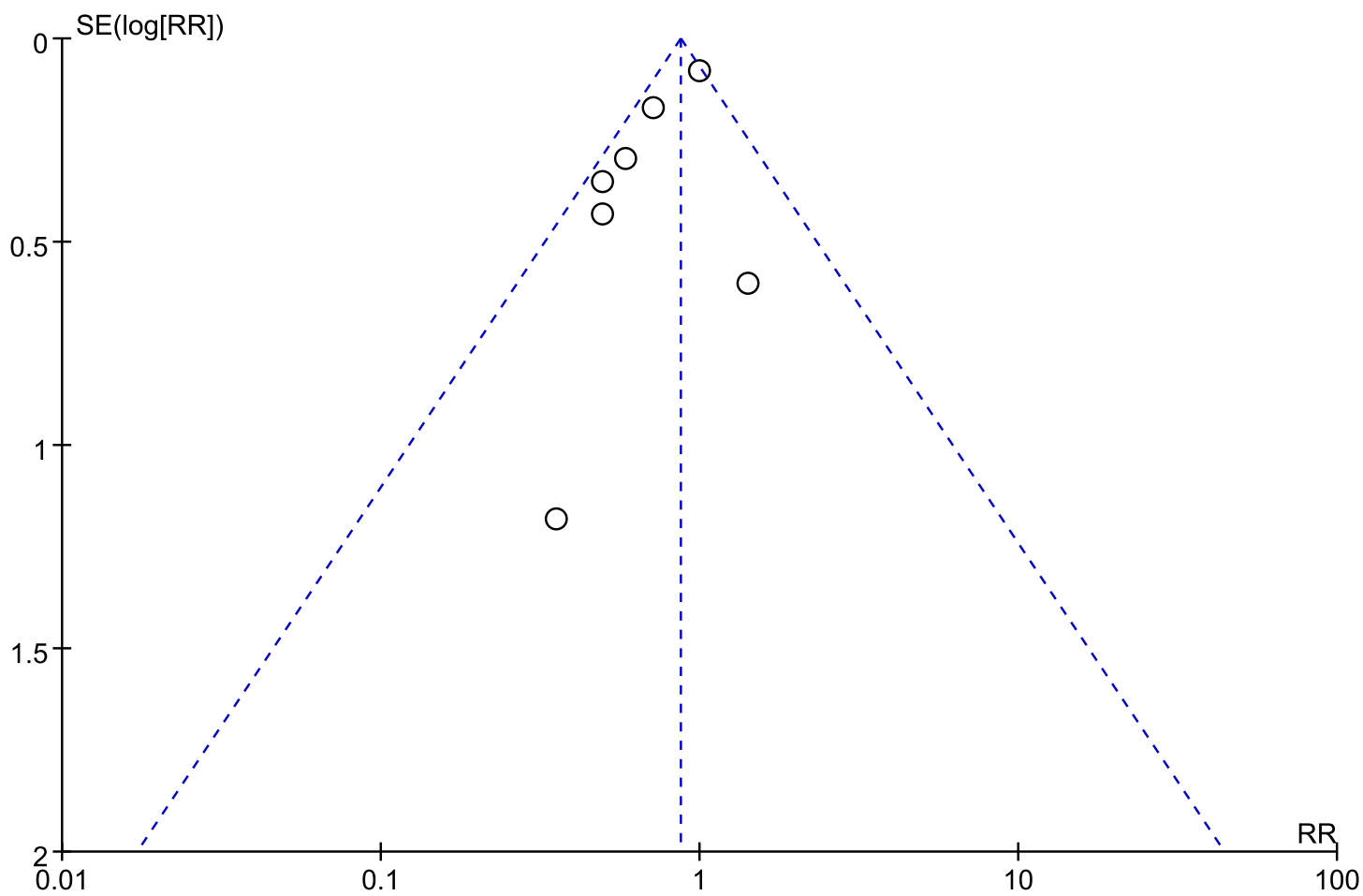

Supplement: Supplementary file 7 — Additional file 7. Publication bias by funnel plot. [file 13054_2020_2765_MOESM7_ESM.pdf]

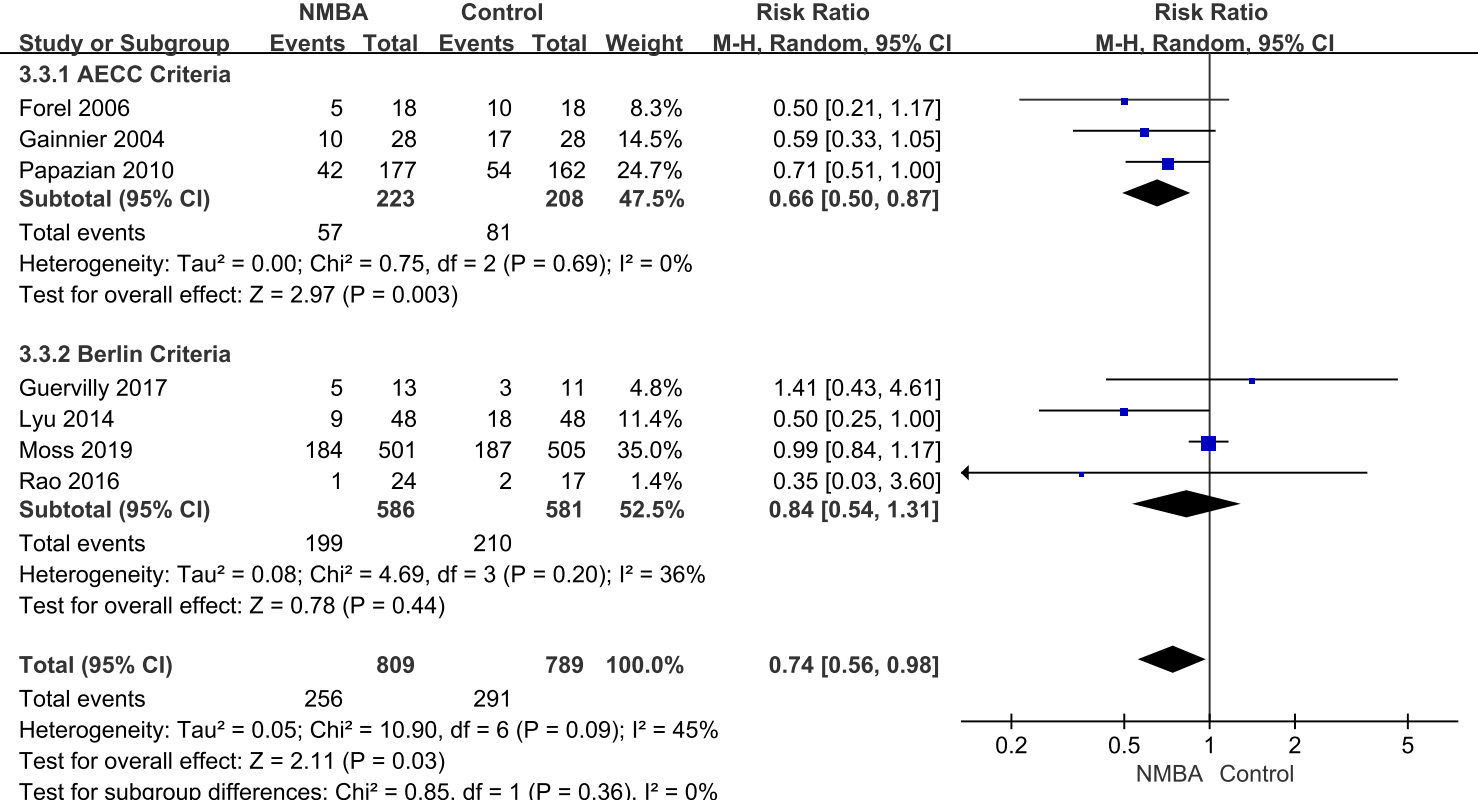

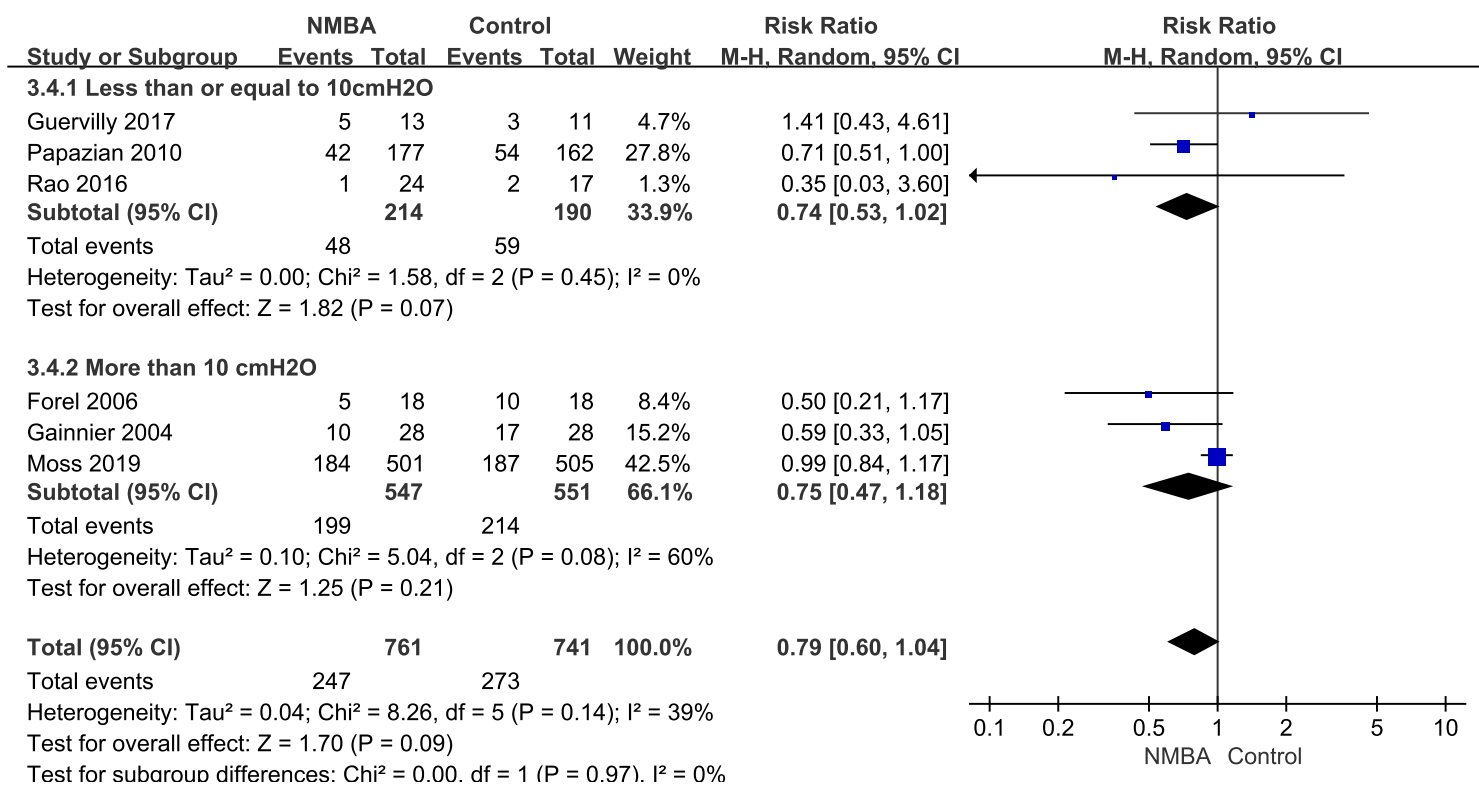

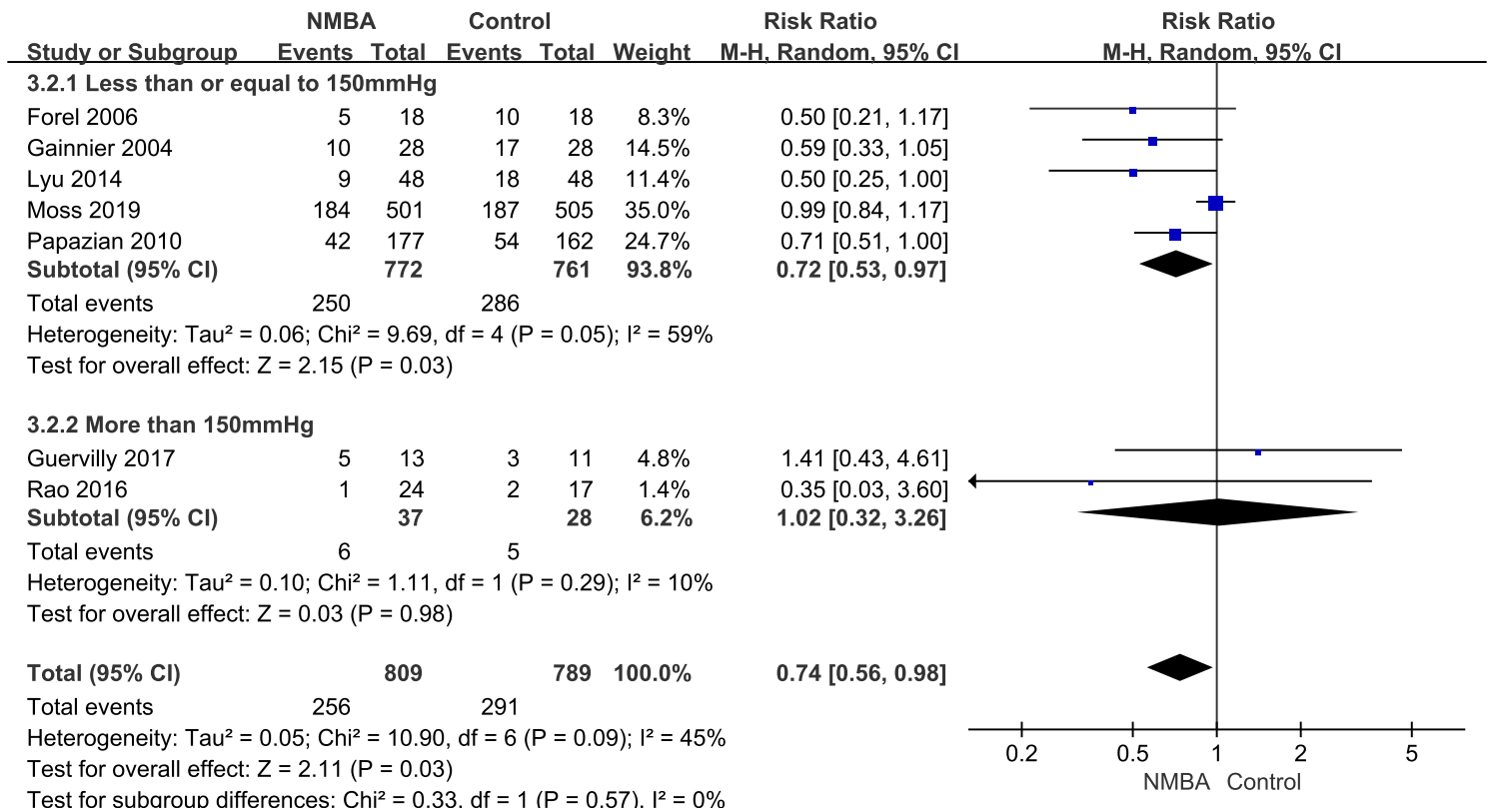

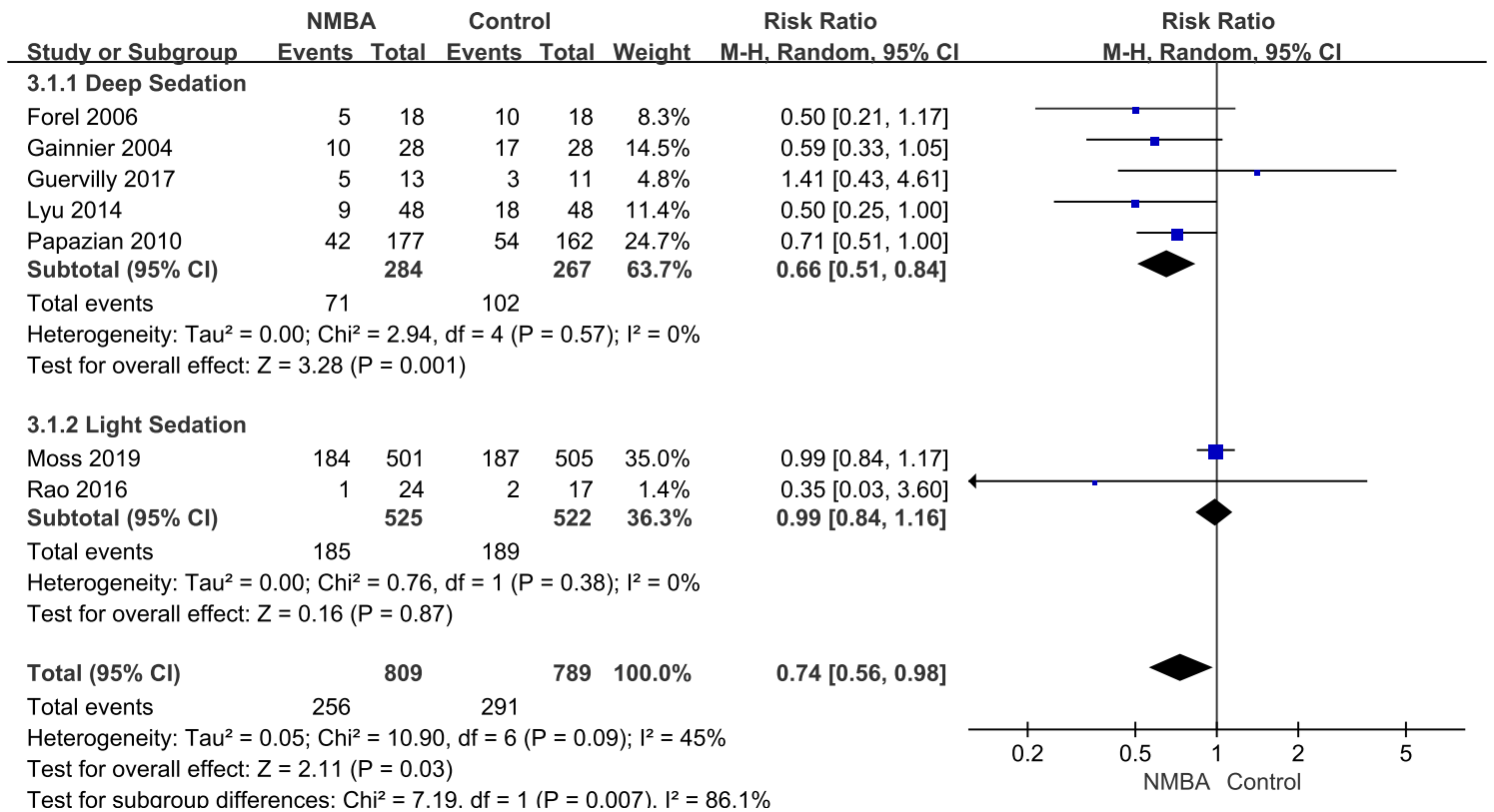

Supplement: Supplementary file 9 — Additional file 9. Sub-group analysis. Patients were divided by ARDS definition (AECC or Berlin), initial PEEP setting (<= 10 mmHg vs > 10mmHg), PaO2 to FiO2 ratio at enrollment (<= 150mmHg vs > 150mmHg) and sedation strategy (light vs deep sedation). [file 13054_2020_2765_MOESM9_ESM.pdf]
