# Supplementary material for: Validation of neuromuscular blocking agent use in acute respiratory distress syndrome: a meta-analysis of randomized trials
Source: Crit Care. 2020 Feb 17;24:54. doi: 10.1186/s13054-020-2765-2 (PMC7027110; doi:10.1186/s13054-020-2765-2)
Supplement: Supplementary file 6 — Additional file 6. Sensitivity analysis. [file 13054_2020_2765_MOESM6_ESM.docx]

# Sensitivity analysis

## Mortality (truncated to day-28) sensitive analysis

| 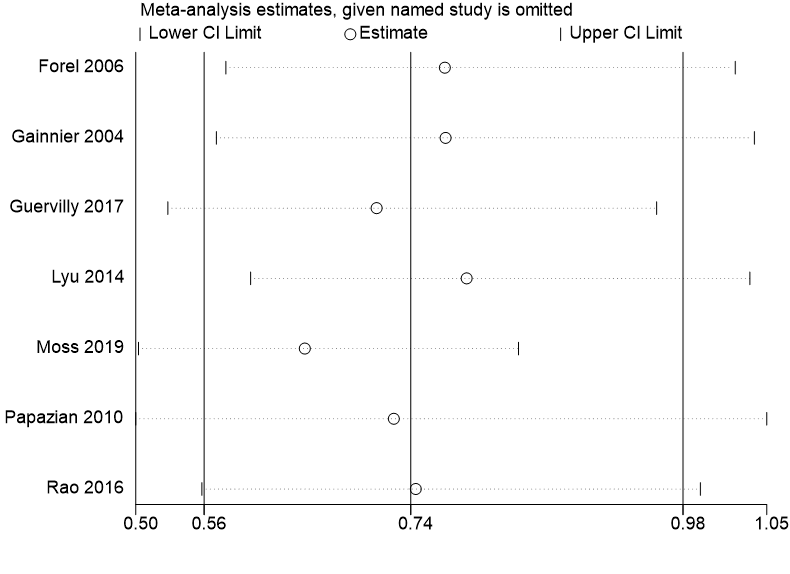 | | | |
| --- | --- | --- | --- |
| **Study omitted** | **Estimated RR** | **[95% Conf. Interval]** | |
| Forel 2006 | 0.77 | 0.58 | 1.02 |
| Gainnier 2004 | 0.77 | 0.57 | 1.04 |
| Guervilly 2017 | 0.71 | 0.53 | 0.96 |
| Lyu 2014 | 0.79 | 0.60 | 1.04 |
| Moss 2019 | 0.65 | 0.51 | 0.84 |
| Papazian 2010 | 0.73 | 0.50 | 1.05 |
| Rao 2016 | 0.75 | 0.56 | 0.99 |
| Combined | 0.74 | 0.56 | 0.98 |

## Mortality (truncated to day-90) sensitive analysis

| 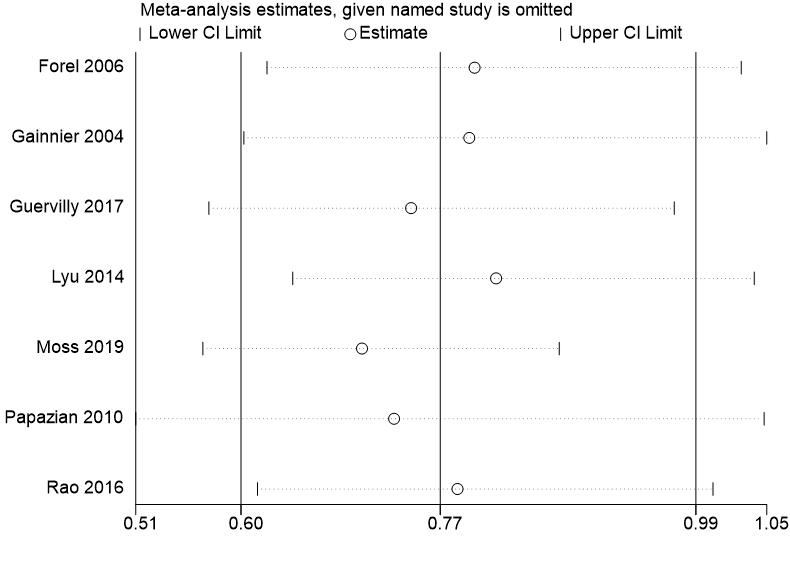 | | | |
| --- | --- | --- | --- |
| **Study omitted** | **Estimated RR** | **[95% Conf. Interval]** | |
| Forel 2006 | 0.80 | 0.62 | 1.03 |
| Gainnier 2004 | 0.79 | 0.60 | 1.05 |
| Guervilly 2017 | 0.75 | 0.57 | 0.97 |
| Lyu 2014 | 0.82 | 0.64 | 1.04 |
| Moss 2019 | 0.70 | 0.57 | 0.87 |
| Papazian 2010 | 0.73 | 0.51 | 1.05 |
| Rao 2016 | 0.78 | 0.61 | 1.00 |
| Combined | 0.77 | 0.60 | 0.99 |
