# Supplementary material for: Validation of neuromuscular blocking agent use in acute respiratory distress syndrome: a meta-analysis of randomized trials
Source: Crit Care. 2020 Feb 17;24:54. doi: 10.1186/s13054-020-2765-2 (PMC7027110; doi:10.1186/s13054-020-2765-2)
Supplement: Supplementary file 8 — Additional file 8. Univariate meta-regression analysis. Following factors were used for meta-regression, publishing year, sample size, language (English or Chinese), ARDS definition (AECC or Berlin criteria), estimated average PaO2 to FiO2 ratio of ARDS patients at enrollment, estimated average improvement of PaO2 to FiO2 ratio 24 hours after enrollment, quality of the study, percentage of patients with intra-pulmonary etiology, sedation strategy (light or deep). [file 13054_2020_2765_MOESM8_ESM.pdf]

Following factors were concluded in the univariate meta-regression analysis,

- Year when the study was published,
- No. of subjects included in the study,
- Language, English or Chinese,
- ARDS diagnosis criteria, AECC or Berlin criteria,
- Estimated average PaO_2 to FiO_2 ratio of ARDS patients at enrollment
- Estimated average improvement of PaO_2 to FiO_2 ratio 24 hours after intervention.
- Quality of the study. The quality of the study was quantified as 'Yes' as 2 points, 'Unclear' as 1 point and 'No' as 0 point evaluated according to the Cochrane Collaboration's protocols. The scores were then summed up,
- Percentage of patients with ARDS from intra-pulmonary origins,
- Sedation strategies, as deep sedation (with RASS score of 6) or light sedation (with RASS score 0 to -1 or Ramsay score 2 to 4)
- Estimated average PEEP value at the initial setting

Study	Year	Subject	Language	Criteria	P/F ratio	P/F ratio change	Score	lung origin (%)	Sedation	PEEP (mmHg)
Forel et al	2006	36	English	AECC	117	50	9	0.81	Deep	12.1
Gainnier et al	2004	56	English	AECC	124.5	27.5	10	0.8	Deep	11
Guervilly et al	2017	24	English	Berlin	154	NR	9	0.88	Deep	9
Lyu et al	2014	96	Chinese	Berlin	142	56	10	NR	Deep	NR
Moss et al	2019	1006	English	Berlin	98.5	77.85	12	0.75	Light	12.55
Papazian et al	2010	339	Chines	AECC	110	55.5	14	0.78	Deep	9.2
Rao et al	2016	41	English	Berlin	170	NR	8	0.56	Light	7

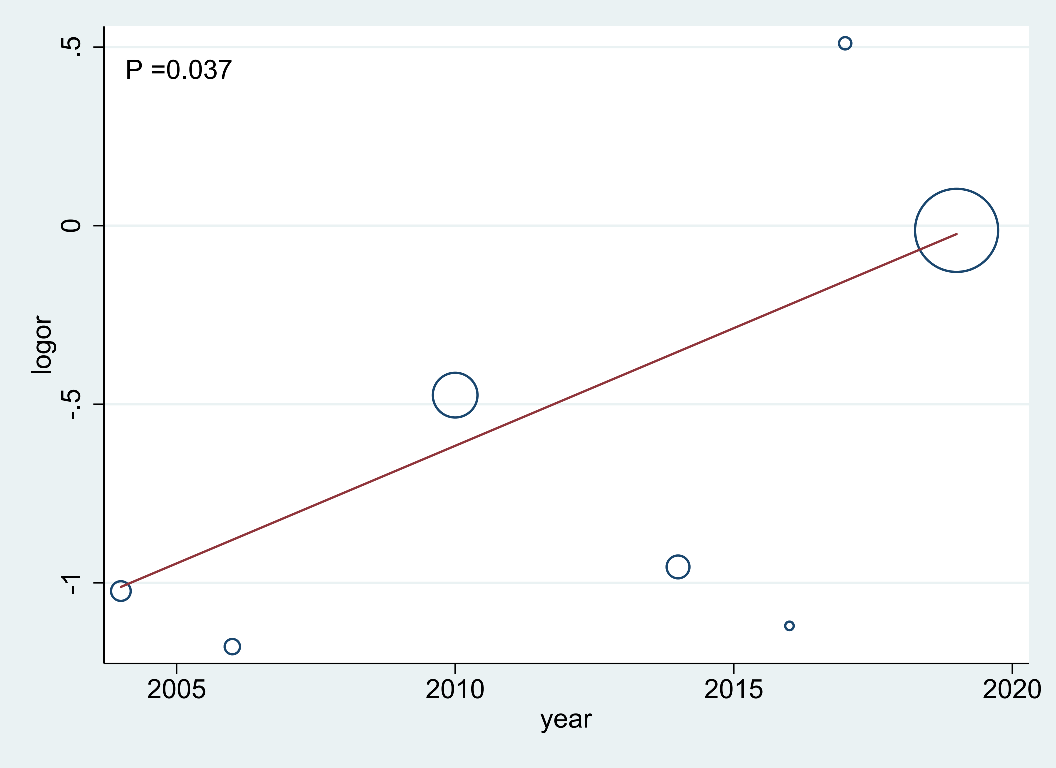
P =0.037
.5
0
-.5
-1
logor
2005
2010
2015
2020
year

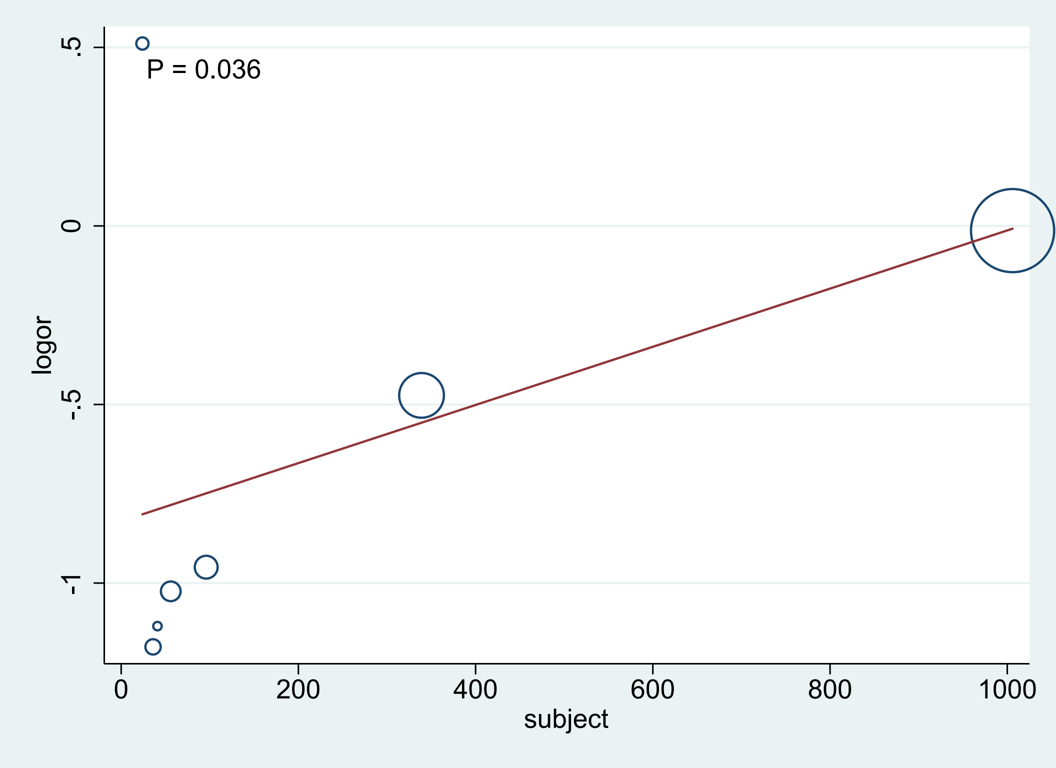
P = 0.036
logor
.5
0
-.5
-1
0
200
400
600
800
1000
subject

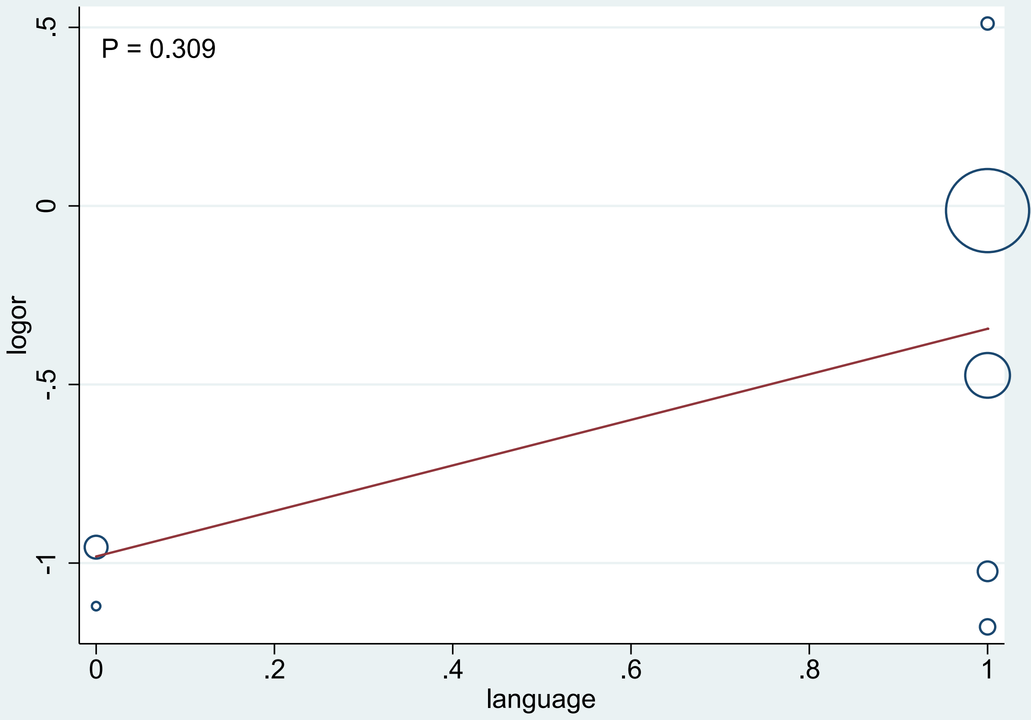

P = 0.309
.5
0
-.5
-1
logor
0
.2
.4
.6
.8
1
language

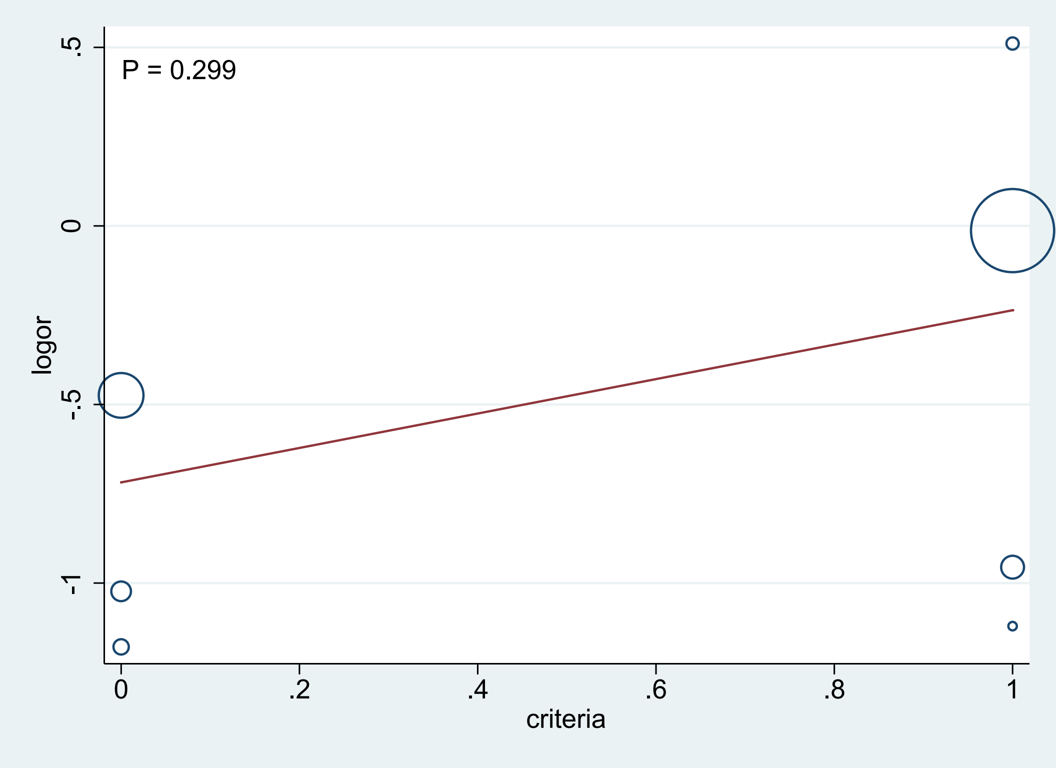

P = 0.299
logor
.5
0
-.5
-1
0
.2
.4
.6
.8
1
criteria

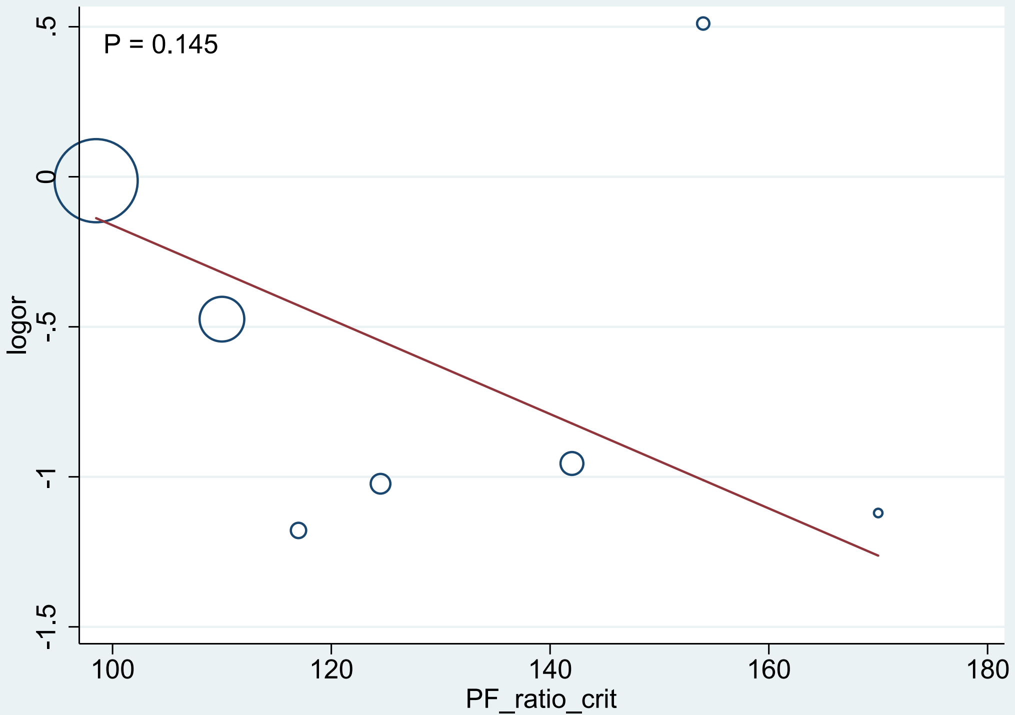
P = 0.145
logor
.5
0
-.5
-1
-1.5
100
120
140
160
180
PF_ratio_crit

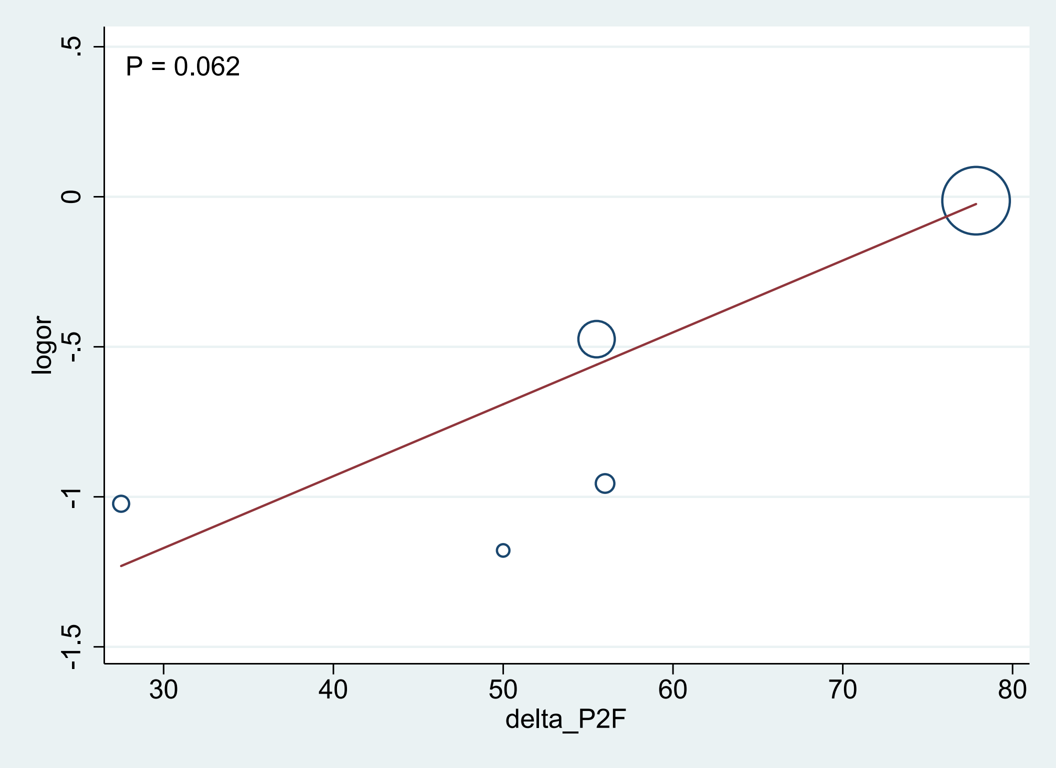
P = 0.062
logor
.5
0
-.5
-1
-1.5
30
40
50
60
70
80
delta_P2F

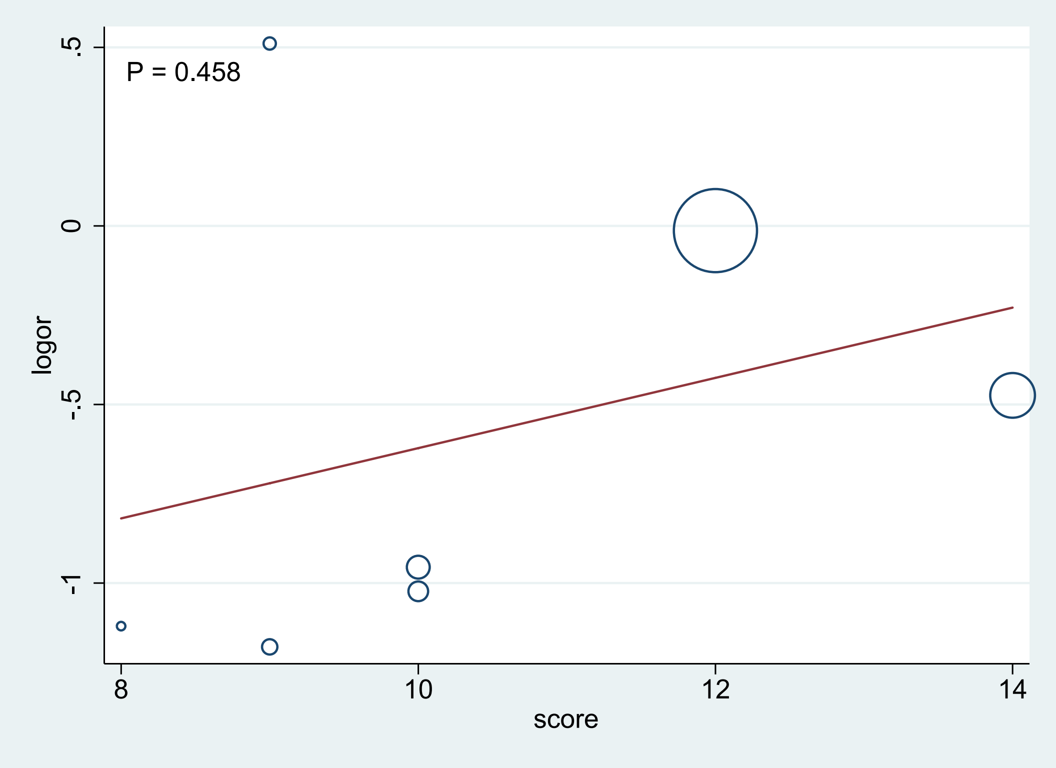
P = 0.458
logor
.5
0
-.5
-1
8
10
12
14
score

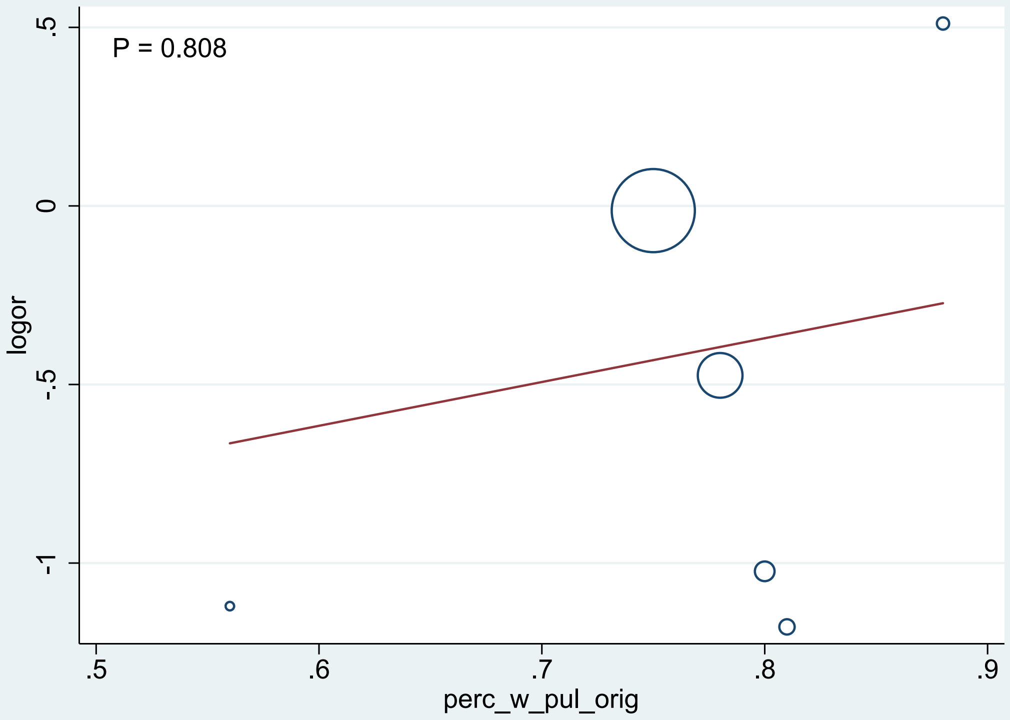

P = 0.808
logor
.5
0
-.5
-1
.5
.6
.7
.8
.9
perc_w_pul_orig

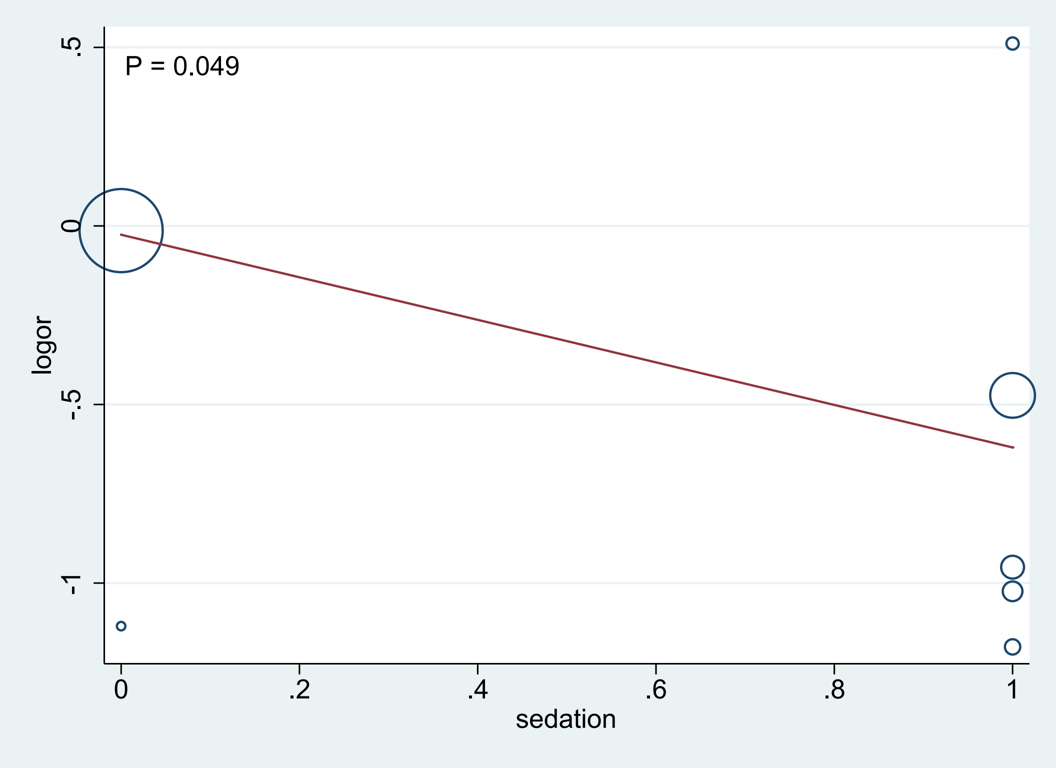

P = 0.049
.5
0
-.5
-1
logor
0
.2
.4
.6
.8
1
sedation

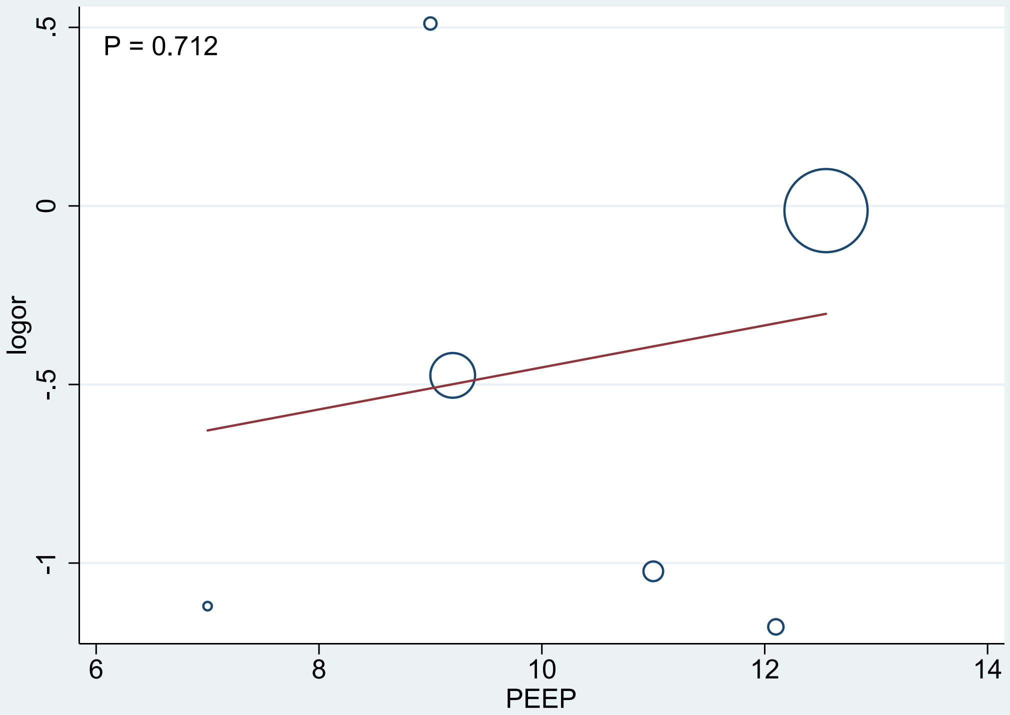
P = 0.712
.5
0
-.5
-1
logor
6
8
10
12
14
PEEP
